# Deficiency of the X-inactivation escaping gene *KDM5C* in clear cell renal cell carcinoma promotes tumorigenicity by reprogramming glycogen metabolism and inhibiting ferroptosis

**DOI:** 10.7150/thno.60233

**Published:** 2021-08-04

**Authors:** Qian Zheng, Pengfei Li, Xin Zhou, Yulong Qiang, Jiachen Fan, Yan Lin, Yurou Chen, Jing Guo, Fan Wang, Haihua Xue, Jie Xiong, Feng Li

**Affiliations:** 1 Department of Medical Genetics, School of Basic Medical Sciences, Wuhan University, Wuhan 430071, China.; 2 Department of Gynecology and Obstetrics, Zhongnan Hospital of Wuhan University, Wuhan 430072, China.; 3 Department of Immunology, School of Basic Medical Sciences, Wuhan University, Wuhan 430071, China.; 4 Hubei Provincial Key Laboratory of Allergy and Immunology, Wuhan 430071, China.

**Keywords:** KDM5C, glycogen, male predominance, X-inactivation escaping gene, pentose phosphate pathway

## Abstract

**Background:** Clear cell renal cell carcinoma (ccRCC) is characterized by glycogen-laden, unexplained male predominance, and frequent mutations in the Von Hippel-Lindau (*VHL*) gene and histone modifier genes. Besides, poor survival rates of ccRCC patients seem to be associated with up-regulation of the pentose phosphate pathway (PPP). However, the mechanism underlying these features remains unclear.

**Methods**: Whole exome sequencing was used to identify the gene mutation that implicated in the rewired glucose metabolism. RNA-seq analyses were performed to evaluate the function of KDM5C in ccRCC. Furthermore, heavy isotope tracer analysis and metabolites quantification assays were used to study how KDM5C affects intracellular metabolic flux. To provide more *in vivo* evidence, we generated the *Kdm5c*^-/-^ mice by CRISPR-Cas9 mediated gene knockout and performed the xenografts with KDM5C overexpressing or depleted cell lines.

**Results:** A histone demethylase gene *KDM5C*, which can escape from X-inactivation and is predominantly mutated in male ccRCC patients, was identified to harbor the frameshift mutation in the ccRCC cell line with the highest glycogen level, while the restoration of KDM5C significantly reduced the glycogen level. Transcriptome and metabolomic analysis linked KDM5C to metabolism-related biological processes. KDM5C specifically regulated the expression of several hypoxia-inducible factor (HIF)-related genes and Glucose-6-phosphate dehydrogenase (G6PD) that were involved in glycogenesis/glycogenolysis and PPP, respectively, mainly through the histone demethylase activity of KDM5C. Depletion of KDM5C increased the production of glycogen, which was then directed to glycogenolysis to generate glucose-6-phosphate (G6P) and subsequently PPP to produce nicotinamide adenine dinucleotide phosphate hydride (NADPH) and glutathione (GSH), thus conferring cells resistance to reactive oxygen species (ROS) and ferroptosis. KDM5C re-expression suppressed the glucose flux through PPP and re-sensitized cancer cells to ferroptosis. Notably, *Kdm5c*-knockout mice kidney tissues exhibited elevated glycogen level, reduced lipid peroxidation and displayed a transformation of renal cysts into hyperplastic lesions, implying a cancer-protective benefit of ferroptosis. Furthermore, KDM5C deficiency predicted the poor prognosis, and clinically relevant *KDM5C* mutants failed to suppress glycogen accumulation and promoted ferroptosis as wild type.

**Conclusion:** This work revealed that a histone modifier gene inactive mutation reprogramed glycogen metabolism and helped to explain the long-standing puzzle of male predominance in human cancer. In addition, our findings may suggest the therapeutic value of targeting glycogen metabolism in ccRCC.

## Introduction

Clear cell renal cell carcinoma (ccRCC) is the most common form of renal cancer with unexplained male predominance. Its histological feature is an increase in lipid and glycogen-rich cytoplasmic deposits 1, 2, which leads to the concept of ccRCC as a metabolic disease 3-5. However, the role of enriched glycogen in ccRCC has not been fully understood, and the mechanism of glycogen accumulation remains unclear.

The general mechanisms of glycogen synthesis and degradation are the same in all tissues, and the key factors usually involved including phosphoglucomutase 1 (PGM1), glycogen synthase (GYS), protein phosphatase 1 regulatory 3 (PPP1R3) family proteins, and glycogen phosphorylase (GP). The regulation of glycogen metabolism may differ between normal and malignant cells 6. Oncogenes (e.g. MYC), tumor suppressors, and microenvironmental factors (e.g. hypoxia) have been reported to regulate glycogen metabolism in cancer cells 6. Elevated glycogen may be directed to glycogenolysis, not only to enable cancer cells to confront glucose deprivation, but more importantly, to produce G6P and activate the subsequent PPP that generates abundant NADPH 7, which is essential for protecting cells from ROS. Therefore, altered glycogen metabolism might serve as a therapeutic target for ccRCC. Unfortunately, the genetic factors for glycogen accumulation and associated oncogenic functions in ccRCC are not fully understood.

The genetic changes that drive ccRCC mainly include alterations in *VHL*, which is mutated in 80% of ccRCC to control the cellular oxygen sensing 8. Under normoxic conditions, the loss of VHL will result in constitutive activation of HIF and up-regulation of HIF target genes. VHL reconstituted cells exhibits reduced lipid storage and became insensitive to the induction of ferroptosis 9. Although VHL loss leads to glycogen accumulation 10, it cannot explain why almost all ccRCCs (including VHL proficient samples) exhibit a glycogen-laden phenotype. Furthermore, VHL deficiency has not been shown to cause G6P up-regulation and PPP activation, suggesting that unknown factors are required for the rewired glycogen metabolism in ccRCC.

The systemic sequencing has shown that ccRCC harbors mutations in a number of chromatin modifier genes, including *PBRM1, SETD2, BAP1,* and *KDM5C* 11, 12. Given that ccRCC is a metabolic disease, it would be very interesting and necessary to analyze the potential roles of mutated chromatin modifier genes in the metabolic remodeling of ccRCC. As a tumor suppressor, the *KDM5C* mutation rate is 18% in ccRCC 13 and its loss-of-function mutations are more frequently identified in males 14. Furthermore, the *KDM5C* gene is located on the X chromosome and has been reported to escape from X-inactivation, resulting in higher mRNA expression in female tissues 14. Although KDM5C is assumed to protect females from the onset of cancer and has been shown to affect the instability of the heterochromatin genome in ccRCC 15, its exact role in ccRCC is still largely unknown.

Here, we demonstrated that KDM5C synergistically regulated glycogenesis, glycogenolysis, and PPP, most likely through its histone demethylase activity. The metabolic changes caused by KDM5C deficiency altered the response of tumor cells to ROS and ferroptosis inducers, thereby enhancing the tumorigenicity. These findings suggested that loss of X-inactivation escaping gene *KDM5C* leaded to cancer metabolic reprogramming and might contribute to the male predominance in ccRCC.

## Results

### The ccRCC cell line with the highest glycogen level harbors the frame-shift mutation in *KDM5C* gene

**Figure 1:**
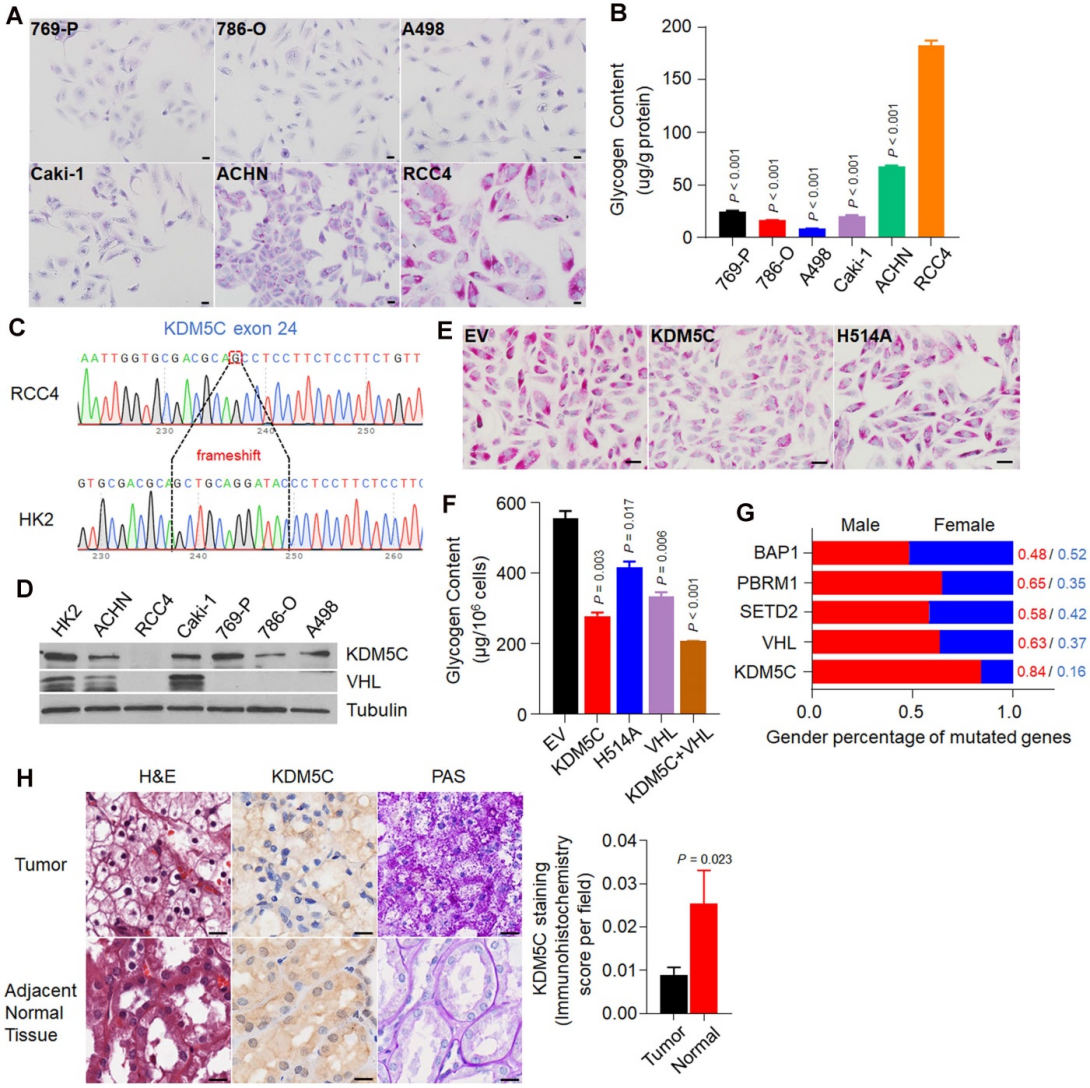
** X-inactivation escaping gene *KDM5C* harbors frame-shift mutation in ccRCC cell line with the highest glycogen level. A,** Periodic acid-schiff (PAS) staining of a series of ccRCC cell lines. Scale bar, 20 µm. **B,** The glycogen level determination of indicated cell lines. **C,** The frame-shift mutation of KDM5C identified in RCC4 exome sequencing compared with HK2. **D,** The KDM5C and VHL protein level detected by Western Blot in various ccRCC cell lines. **E,** PAS staining of stable cell lines including RCC4-EV, RCC4-KDM5C, RCC4-H514A. Scale bar, 20 µm. **F,** Bar graph showing the intracellular glycogen content in indicated cells. **G,** Stacked bar graph showing the gender ratio of frequently mutated genes in KIRC. **H,** Representative hematoxylin and eosin (H&E) stained (left), KDM5C immunohistochemically stained (middle), and PAS stained (right) sections from the tumor (top) and adjacent normal tissue (bottom). Scale bar, 40 µm. Bar graph showing the statistical analysis of indicated tissues stained with KDM5C antibody. *P* values were calculated using two-tailed Student's t-test. Data represent means ± SD. Data are representative of three independent experiments.

To investigate if there any frequently mutated gene in ccRCC is associated with the formation of glycogen laden in ccRCC, we first screened a series of ccRCC cell lines using Periodic Acid-Schiff (PAS) staining. As shown in **Figure 1A**, among the six cell lines, RCC4 exhibited the highest glycogen level, which was then confirmed by glycogen quantification (**Figure 1B**). Our observation was consistent with the previous study that RCC4 cell line was rich in glycogen 16. Except for ACHN and Caki-1 cells, all other cell lines are VHL deficient, suggesting that additional factors are required for the extremely high glycogen level in RCC4.

To identify additional factors, we performed exon sequencing with RCC4 and HK-2 cells (**Figure S1A**). We chose HK-2 cells as a control because HK-2 cells were derived from proximal tubule epithelial cells, where ccRCCs appear. As shown in **Table 1**, the sequencing identified a number of genomic alterations in RCC4 cells, including *KDM5C* and *VHL*, which had the frame-shift and missense mutations, respectively (**Figure 1C and Figure S1B**). Western blot results from multiple cell lines showed that both KDM5C and VHL proteins were absent in RCC4 cells (**Figure 1D**). Therefore, our work provided for the first time convincing genomic evidence that high glycogen levels in cancer cells might be associated with an inactive *KDM5C* mutation.

We then examined whether restoration of KDM5C would reduce the glycogen level in RCC4 cells. RCC4 cell lines stably expressing the wild-type KDM5C or its enzymatically inactive mutant H514A were constructed (**Figure S1C**), and PAS staining results demonstrated that wild-type KDM5C but not its mutant efficiently reduced the glycogen level (**Figure 1E**), which was confirmed by the glycogen quantification assay (**Figure 1F**). Consistent with previous study, re-expression of VHL reduced glycogen level as well. To further confirm that if the effect of KDM5C and VHL on glycogen was independent of each other, we constructed a RCC4 cell line that stably expressed both KDM5C and VHL. Restoration of these two proteins moderately drove glycogen levels further **(Figure 1F)**, implying that KDM5C and VHL might play partially overlapping role in regulating glucose metabolism. These results further supported the notion that *KDM5C* mutation promote the formation of glycogen laden in ccRCCs.

As an X-inactivation escaped gene, our analysis revealed a lower KDM5C expression in normal tissues of male Kidney Renal Clear Cell Carcinoma (KIRC) patients (**Figure S1D**). ccRCC is characterized by male predominance. The RCC4 cell line used here was also derived from a male patient. Among genes that were frequently mutated in ccRCC, the *KDM5C* mutation exhibited the highest male to female ratio (**Figure 1G**). We then performed KDM5C immunohistochemistry (IHC) using the ccRCC tissue chip. The KDM5C protein level was higher in female normal tissues than that in male (**Figure S1E**), but it decreased in ccRCC patients, accompanied by a significant increase in glycogen (**Figure 1H**). Therefore, understanding the exact role of KDM5C in ccRCC may help explain the male dominance of ccRCC.

### KDM5C deficiency elicits ccRCC-specific metabolic phenotypes

**Figure 2:**
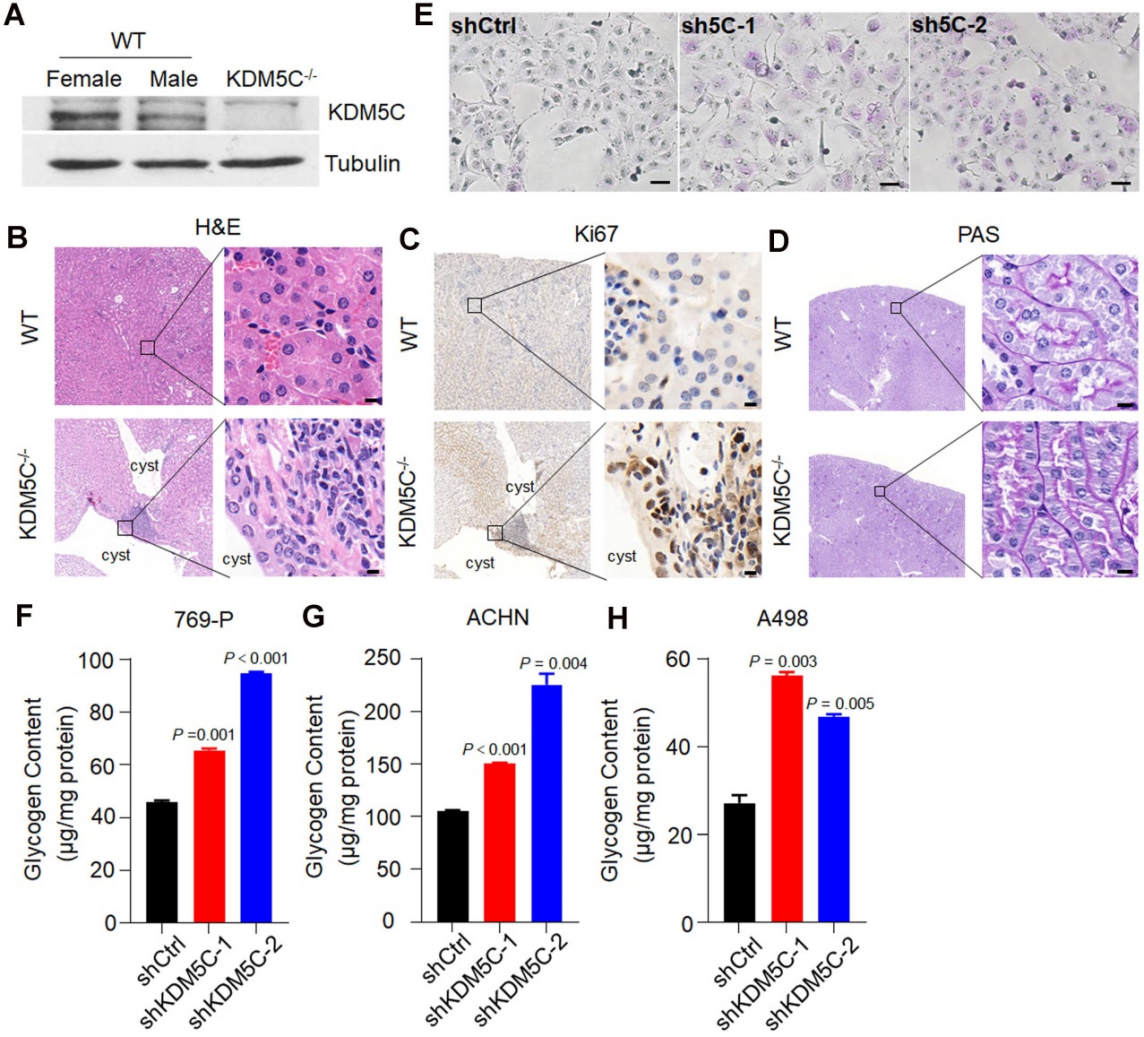
***kdm5c* knock out elicit ccRCC-specific metabolic phenotype. A,** Western blot analysis of the KDM5C protein level in renal tissues of wild-type and *Kdm5c*^-/-^ mice. **B-D,** Hematoxylin and eosin (B), Ki67 immunohistochemical (C), and PAS (D) staining in the renal cortex from indicated mice, Scale bar, 20 µm. **E,** PAS staining of indicated 769-P cell lines. **F-H,** The intracellular glycogen content of indicated engineered 769-P (F), ACHN (G), A498 (H) cell lines. *P* values were calculated using paired t-test. Data represent means ± SD. Data are representative of three independent experiments.

To provide more *in vivo* evidence that KDM5C deficiency was important for ccRCC initiation and glycogen accumulation, we generated the *Kdm5c*^-/-^ mice by CRISPR-Cas9 mediated gene knockout (**Figure S2A**). The mice were sacrificed at 10.0 ± 1.0 months of age and analyzed for the putative abnormalities of the kidney. As a gene that escapes X-inactivation, the KDM5C protein level in the female kidney was higher than that in males and was undetectable in *Kdm5c*^-/-^ mice (**Figure 2A**). Although the loss of KDM5C did not cause obvious renal carcinomas, renal cysts were observed in 50% of mice with a *KDM5C* deletion but was not found in any control mice (**Figure 2B**). To examine whether the renal cyst contained hyperproliferative areas, we performed the Ki67 IHC. As shown in **Figure 2C**, a number of epithelial cells facing the lumen of renal cysts showed strong Ki67 staining, while the renal tissue of control mice barely showed the Ki67 signal. Importantly, as assessed by PAS staining, the renal tubules of *Kdm5c*^-/-^ mice displayed elevated accumulation of cytoplasmic glycogen **(Figure 2D)**. These observations suggested that KDM5C deficiency might elicit ccRCC-specific metabolic phenotypes and promote tumor formation. Moreover, we generated KDM5C knocked down stable cell lines, either PAS staining in 769-P cells (**Figure 2E**) or glycogen quantification assay in multiple ccRCC cell lines indicated an increased glycogen level upon KDM5C depletion (**Figure 2F-2H, Figure S2B**). We then knocked down VHL and KDM5C individually or together in Caki-1 cells (**Figure S2C**), which is a ccRCC cell line that express both VHL and KDM5C proteins. Depletion of any one of them lead to increased glycogen level, while knocking down them together exhibited an additive effect, which was consistent with the result of restoration. These results showed that KDM5C might have VHL/HIF independent ability to regulate glycogen.

### KDM5C histone demethylase activity is required for its role in regulating genes expression involved in glycogenesis, glycogenolysis and PPP

**Figure 3:**
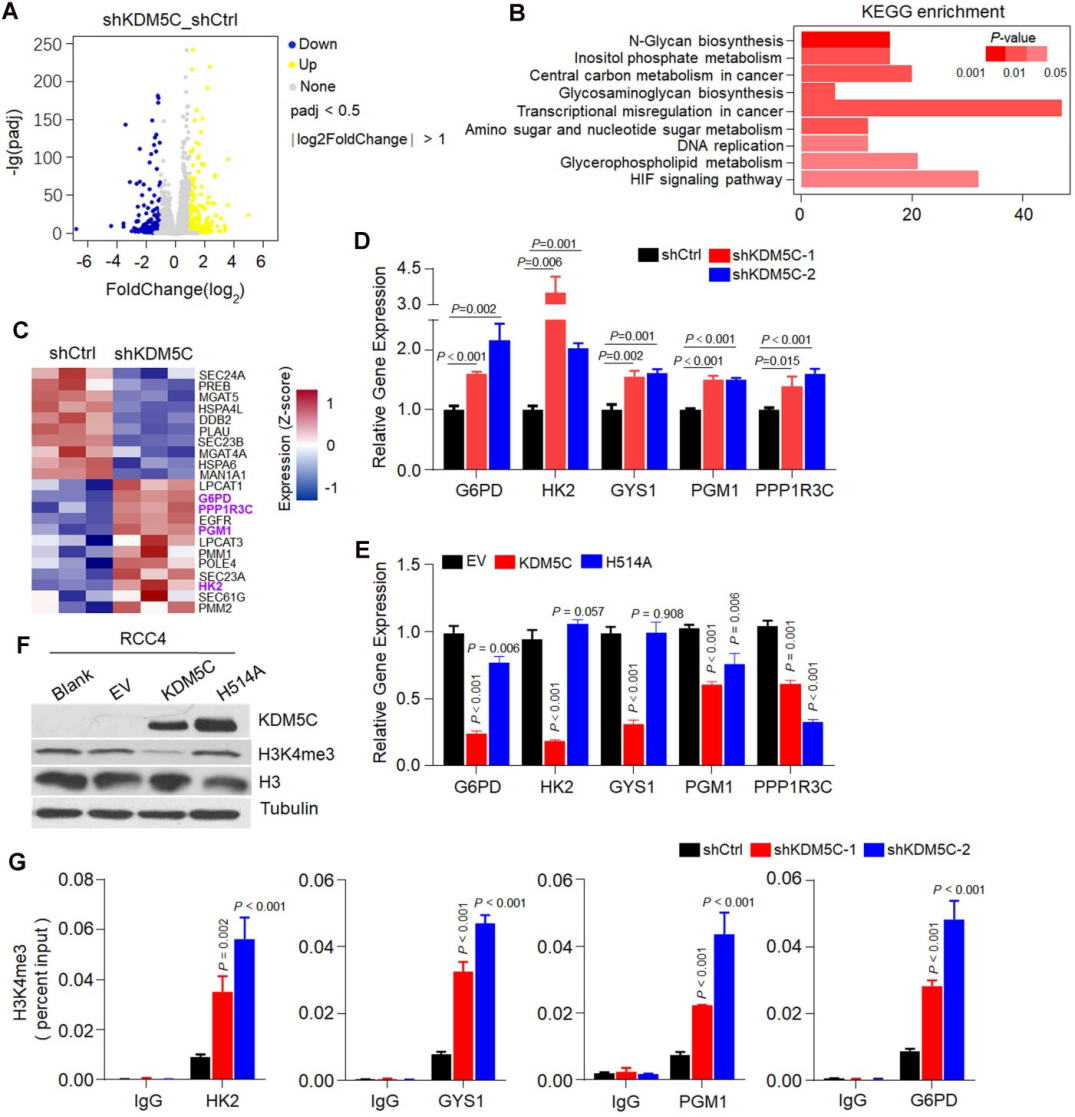
** Histone demethylase activity is required for KDM5C to inhibit glycogen metabolism. A,** Volcano plots of RNASeq data for ACHN cells transfected with shKDM5C or shCtrl. Blue, down-regulated genes; yellow, up-regulated genes. The data of fold changes were derived from indicated RNA-Seq, and the* P*-value was calculated by t-test and adjusted using the False Discovery Rate (FDR) method. **B,** Kyoto Encyclopedia of Genes and Genomes (KEGG) pathway enrichment analysis of the identified differentially expressed genes (DEGs) from the indicated RNASeq data. *P* -value was determined by Fisher exact test. **C,** Heat map depicting the significantly differentially expressed genes between indicated cells. Red indicates high relative expression, and blue indicates low relative expression. Genes involved in glycogen synthesis/degradation and pentose phosphate pathway (PPP) are indicated in purple (three replicates per group). **D-E,** qPCR analysis of the expression of selected genes in the heat map in ACHN (D) or RCC4 (E) stable transfected cells. For statistical plots, the data are shown as the mean ± SD. *P*-value was calculated by the two-tailed Student's t-test. **F,** Western blotting analysis of KDM5C, H3 and H3K4me3 level in indicated cells. **G,** ChIP-qPCR analysis of H3K4me3 showing the increased binding signal on the promoters of* HK2, GYS1, PGM1,* and* G6PD* with KDM5C deficiency in ACHN cells. *P* values were calculated using paired t-test. Data represent means ± SD.

To explore the role of KDM5C in glycogen metabolism, we first performed RNA-seq in ACHN-shKDM5C or RCC4-KDM5C cell lines and the controls (**Figure 3A and Figure S3A**). As shown in **Figure 3B**, KDM5C depletion in ACHN cells caused enriched pathways related to central metabolism, HIF response, amino sugar metabolism, and transcriptional dysregulation in cancer, which were similar to that of KDM5C restoration in RCC4 cells (**Figure S3B**), suggesting that KDM5C was essential for cellular metabolic regulation.

Several genes involved in glycogen synthesis/degradation and subsequent PPP, including *HK2*,* PGM1*, *PPP1R3C*, and* G6PD*, were identified by RNA-seq (**Figure 3C**). KDM5C depletion lead to increased expression of several HIF-related genes, including *HK2*, *GYS1*,* PGM1*, and *PPP1R3C* (**Figure 3D**), implying that KDM5C might suppress the HIF responsive genes in ccRCC cells. In addition, *G6PD*, a key gene controlling glucose flux towards PPP, which has not been reported as a HIF target gene, was also up-regulated, implying that KDM5C deficiency might shunt glucose towards PPP. Accordingly, KDM5C restoration inhibited the expression of *HK2*, *GYS1*,* PGM1*, and *PPP1R3C* (**Figure 3E**). Interestingly, *G6PD* mRNA level was significantly down-regulated by ectopic KDM5C but not VHL (**Figure 3E; Figure S3C**), suggesting that KDM5C and VHL played critical but partially distinct roles in ccRCC metabolic regulations. In contrast, expressing mutant KDM5C H514A with impaired histone demethylase activity failed to suppress the expression of these genes, indicating that histone demethylase activity was required for regulating glycogen metabolism.

We then compared the expression levels of these genes in KIRC and corresponding normal tissues. The results showed that *HK2*,* PGM1*, *PPP1R3C, GYS1*, and* G6PD* were significantly up-regulated in ccRCC tumor tissues (**Figure S3D-H**).

KDM5C can regulate gene expression by modulating the H3K4me3 level of the gene promoter. Re-expression of wild-type KDM5C instead of its H514A mutant reduced the H3K4me3 level (**Figure 3F**). We then performed the ChIP-PCR to measure changes in the H3K4me3 levels of the promoters of those genes. As shown in **Figure 3G**, the depletion of KDM5C resulted in increased levels of H3K4me3 on the promoters of those genes except *PPP1R3C*, indicating that KDM5C was capable of directly regulating the gene expression of *HK2*, *GYS1*,* PGM1*, and *G6PD.* Because H3K4me3 states centered at the transcription start site (TSS) for RNApII transcripts 17, we concluded that loss of KDM5C may promotes glycogen related genes expression through enhancing the H3K4me3 level.

### KDM5C suppresses the flux of glucose to PPP

**Figure 4:**
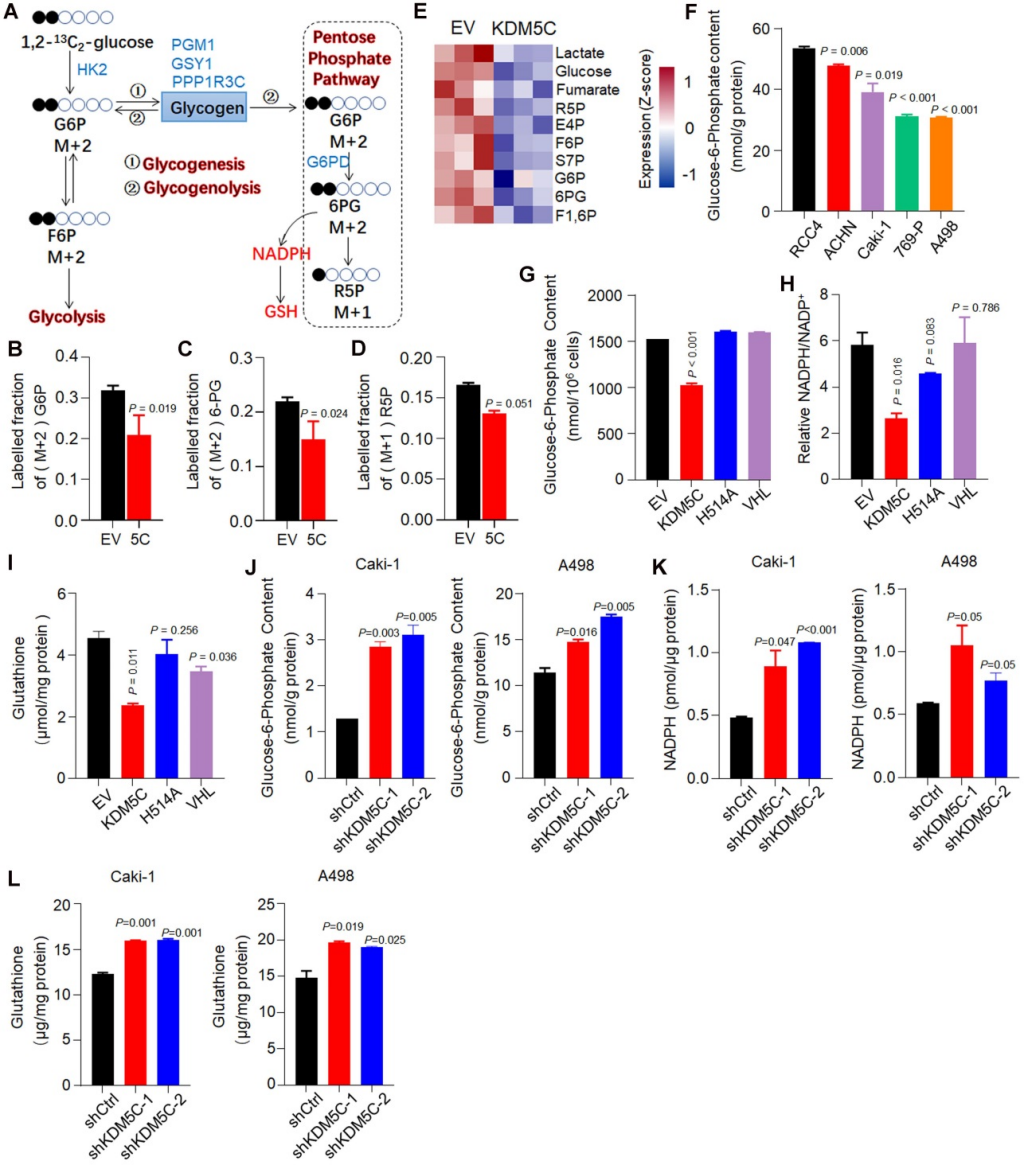
** KDM5C suppresses the glucose flux through the pentose phosphate pathway. A,** Schematic diagram for the conversion of 1,2-^13^C_2_-glucose into various metabolites. The KDM5C regulated genes involved in glycogen metabolism and PPP are indicated in blue. **B-D,** Control or KDM5C-overexpressing RCC4 cells were cultured with 1,2-^13^C_2_-glucose for 24 h (three replicates per group), and LC-MS was performed to determine (M)+2 G6P (B), (M)+2 6-PG (C), and (M)+1 R5P (D), respectively. **E,** Heat map of metabolomics showing the absolute intracellular levels of altered metabolites that were products of pentose phosphate pathway and glycolysis. Red, up-regulated; blue, down-regulated. **F,** The intracellular glucose-6-phosphate (G6P) determination of ccRCC cells. **G-I,** Quantification of intracellular G6P (G), NADPH (H) and glutathione (I) levels in RCC4 derived cell lines. **J-L,** Bar graphs showing intracellular G6P (J), NADPH (K) and glutathione (L) levels in indicated cells. *P* values were calculated using paired t-test. Data represent means ± SD. Data are representative of three independent experiments.

Although KDM5C appeared to simultaneously regulate glycogen synthesis/degradation and the subsequent PPP based on RNA-seq analysis, the direction of the glucose flux regulated by KDM5C remains unclear. We then used a ^13^C carbon tracing method based on liquid-chromatography mass spectrometry to further confirm that KDM5C suppressed the flux of glucose to the PPP (**Figure 4A**). 1,2-^13^C_2_-glucose was added into the medium of RCC4-KDM5C or control cells at the exponential growth phase. As shown in **Figure 4B**, in line with the results of gene expression profiling, the restoration of KDM5C in RCC4 cells resulted in a significant decrease in the mass (M)+2 G6P. Moreover, compared with the control cells, the typical PPP products (M)+2 6-PG and (M)+1 R5P were also decreased (**Figure 4C and 4D**). Because G6P is a major product of glycogenolysis, these results demonstrated that KDM5C suppressed the flux of glucose to PPP.

In addition, we systematically analyzed the absolute levels of intracellular metabolites and verified the data quality by Principal Component Analysis (PCA) (**Figure S4A**). The metabolomics results clearly showed that KDM5C suppressed the production of several products that were involved in PPP and glycolysis (**Figure 4E and Figure S4B**). The isotope tracer analysis also revealed that several glycolysis products, including (M)+2 F6P and (M)+2 lactate, were down-regulated in response to KDM5C overexpression (**Figure S4C**).

Next, we measured the content of several key metabolites in multiple cell lines. Interestingly, similar to glycogen, our results revealed that the G6P level in RCC4 was also higher than other ccRCC cell lines (**Figure 4F**). Wild-type KDM5C, but not the mutant H514A, efficiently reduced the level of G6P and another major PPP product, NADPH (**Figure 4G-H**). Because RCC4 cells also have VHL defects, we then checked whether the loss of VHL had a metabolic alteration similar to KDM5C loss. Although VHL reduced the glycogen content (**Figure 1F**), it failed to decrease the levels of G6P and NADPH, which was consistent with the G6PD mRNA quantification result (**Figure 3E**). These results indicated that inactive mutations of KDM5C and VHL had synergistic but partially distinct roles in ccRCC. Clinical progression of ccRCC is characterized by an increase in glutathione (GSH) 18, an important antioxidant system. The thiol group of GSH reacts with ROS, resulting in the conversion of GSH to glutathione disulfide (GSSG), which can be reduced back to GSH by NADPH. Similar to other metabolites, ectopic KDM5C reduced GSH level (**Figure 4I**).

Consistently, KDM5C deficiency led to increased PPP substrate G6P, PPP product NADPH and GSH in several ccRCC cell lines (**Figure 4J-4L, Figure S4D-4F**). Collectively, our results indicated that KDM5C deficiency in ccRCC cells increased glycogenesis, then directed the resultant glycogen to glycogenolysis to generate G6P and subsequent PPP to generate nucleotide and NADPH.

### KDM5C suppresses tumorigenicity largely by promoting ferroptosis

**Figure 5:**
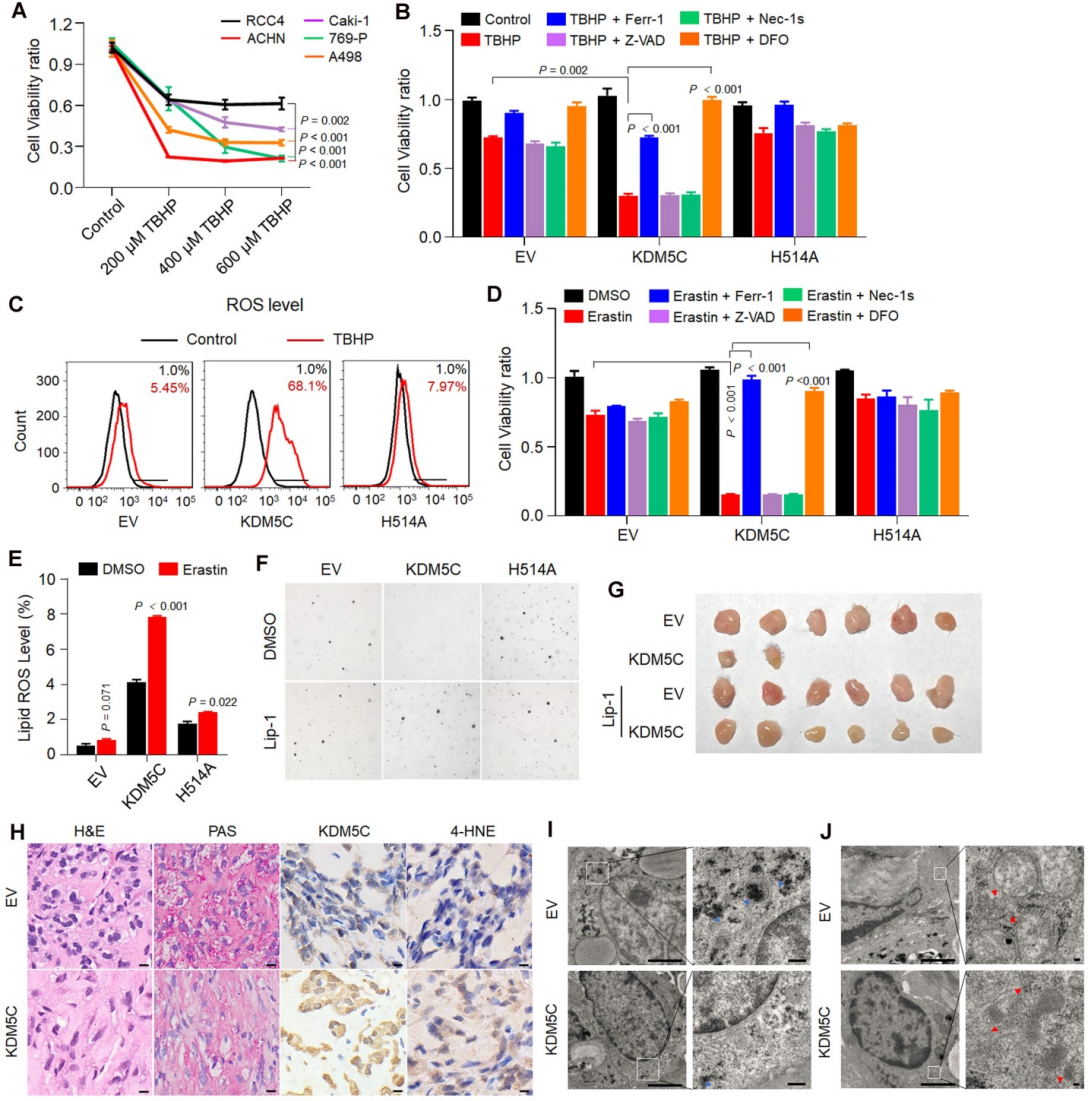
** KDM5C inhibiting tumorigenicity largely by inhibiting ferroptosis. A,** Percentage viability of ccRCC cell lines (ACHN, Caki-1, RCC4, 769-P, A498) following exposure to tert-Butyl hydroperoxide (TBHP) at indicated concentrations for 3 h. *P*-value was calculated by Gehan-Breslow-Wilcoxon test. **B,** Bar graph showing cell viability in indicated cells treated with 200 µM TBHP combined with 5 µM Z-VAD-FMK (Z-VAD), 2 µM Necrostain-1s (Nec-1s), 2 µM Ferrostatin-1 (Ferr-1) or 50 µM deferoxamine (DFO). **C,** Indicated cells were loaded with 500 nM CellROX Green and the ROS were analyzed by flow cytometry. The experiment was repeated in triplicate. **D,** Bar graph showing cell viability in indicated cells treated with 2.5 µM Erastin combined with 5 µM Z-VAD, 2 µM Nec-1s, 2 µM Ferr-1 or 50 µM DFO for 48 h. **E,** After C11-BODIPY staining in indicated cells, lipid peroxidation was assessed by flow cytometry. The experiment was repeated in triplicate. **F,** Soft agar assay of RCC4-EV, RCC4-KDM5C, RCC4-KDM5C-H514A with or without liproxstatin-1(Lip-1). **G,** Image of NOD/SCID mice xenograft tumors derived from RCC4-EV and RCC4-KDM5C cells were treated with 10 mg/kg liproxstatin-1 (Lip-1) or saline, respectively (*n* = 6 per group). **H,** Hematoxylin and eosin (H&E), PAS, and immunohistochemical staining of tumor xenografts derived from RCC4-EV and RCC4-KDM5C cells. Scale bars, 20 µm. **I and J,** Representative images of transmission electron microscopy of indicated tumor xenografts. Blue arrows, glycogen (I); red arrows, mitochondria (J). Scale bars, 2 µm. *P* values were calculated using paired t-test (B, D, E). Data represent means ± SD.

PPP is important for anti-ROS activity and tumor aggressiveness. A recent study has revealed that the major PPP product NADPH is an indicator of ferroptosis 19. Then, we studied the potential role of KDM5C in ROS resistance and ferroptosis. We first compared the ROS resistance by treating various ccRCC cell lines with the ROS inducer *tert*-butyl hydroperoxide (TBHP). As shown in **Figure 5A**, RCC4 exhibited relatively higher ROS resistance than other ccRCC cell lines. After being treated with TBHP, RCC4-KDM5C had more cell death compared to RCC4-H514A cells (**Figure 5B**). Notably, the ROS-induced cell death could be reversed by the ferroptosis inhibitor Ferrostatin-1 (Ferr-1) or the iron chelator deferoxamine (DFO), but not by the apoptosis inhibitor Z-VAD-FMK (Z-VAD) or the necroptosis inhibitor necrostatin-1s (Nec-1s). Consistently, wild-type KDM5C rather than its H514A mutant led to a substantially increased ROS level in the presence of TBHP (**Figure 5C**). Moreover, wild-type KDM5C, but not its H514A mutant, significantly potentiated Erastin-mediated cell death (**Figure 5D**), which could be efficiently reversed by Ferr-1 or DFO, but not by Z-VAD or Nec-1s. Accordingly, increased Erastin-induced lipid peroxidation was more obvious in RCC4-KDM5C cells compared with RCC4-H514A cells (**Figure 5E**). In addition, ectopic KDM5C specifically promoted ROS induced cell death or Erastin-induced ferroptosis in another ccRCC Caki-1 cells (**Figure S5A-C**).

We then studied the role of KDM5C in tumor suppression. Re-expressing wild-type KDM5C, but not its H514A mutant, suppressed the colony formation in RCC4 cells, while treatment with the ferroptosis inhibitor liproxstatin-1 (Lip-1) partially restored colony formation of RCC4-KDM5C cells (**Figure 5F**). Next, we subcutaneously transplanted the tumor cells into NOD/SCID mice. As shown in **Figure 5G**, KDM5C restoration markedly inhibited the RCC4 tumor cell growth in NOD/SCID mice, while the administration of Lip-1 restored the tumor formation. KDM5C expression in xenograft tumors was confirmed by IHC (**Figure 5H**). The PAS staining revealed that xenograft tumors with KDM5C re-expression exhibited a significant reduction in glycogen content (**Figure 5H**), which was further confirmed by electron microscopy (EM) analysis (**Figure 5I**). Ferroptosis is characterized by lipid peroxidation, we therefore performed 4-hydroxynonenal (4-HNE) IHC analysis to measure the lipid peroxidation in RCC4-KDM5C or control cells. Our results showed that increased 4-HNE staining was observed in tumor cells re-expressing KDM5C compared with control cells (**Figure 5H**), confirming the existence of ferroptosis. In addition, the EM analysis revealed that xenograft tumors with KDM5C re-expression contained shrunken mitochondria with increased membrane density (**Figure 5J**), a morphologic feature of ferroptosis.

### KDM5C deficiency confers cancer cells resistance to ROS and ferroptosis

**Figure 6:**
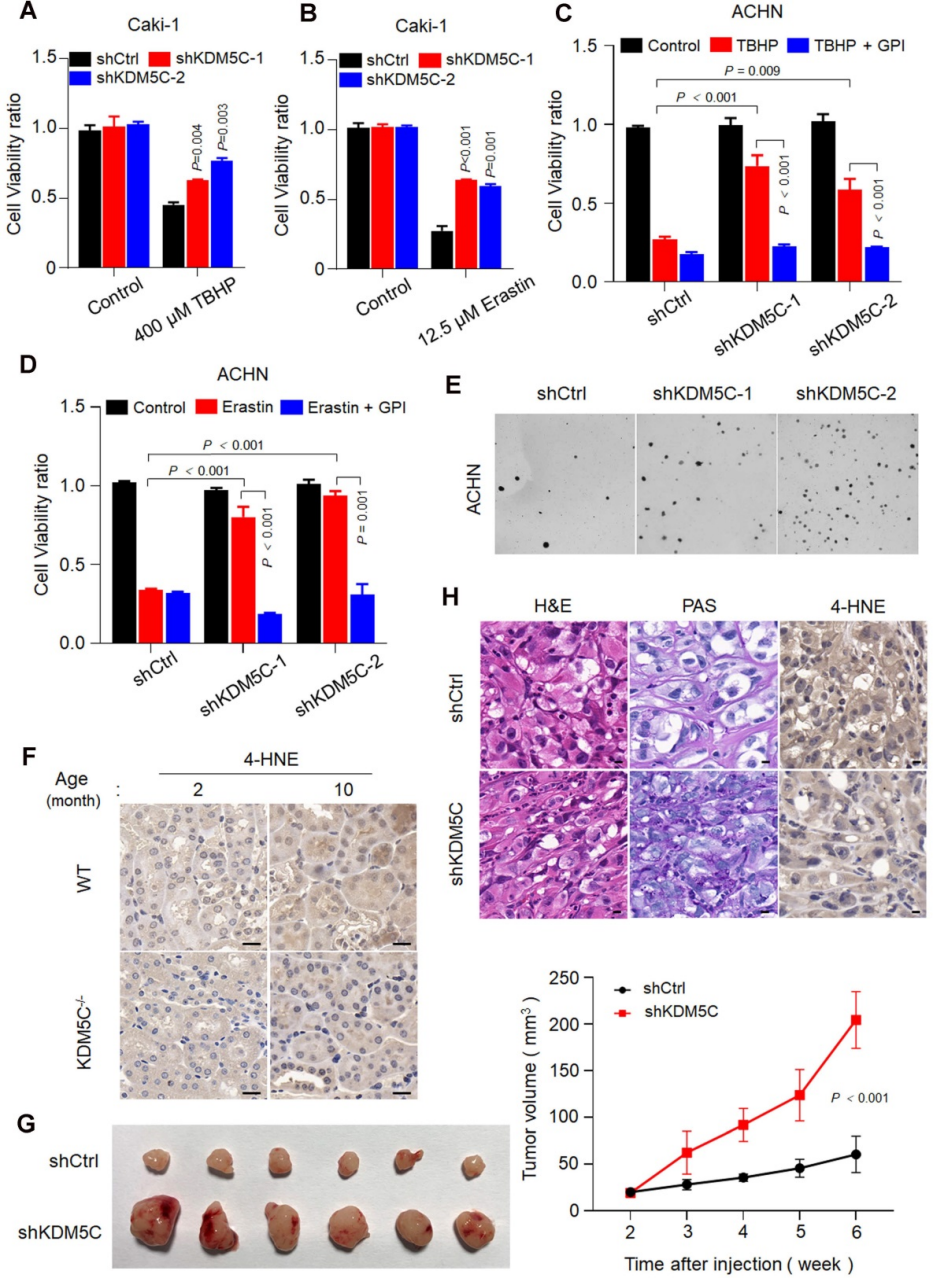
** Loss of KDM5C renders both aged kidney cell and ccRCC cells resistant to ferroptosis. A-B,** Cell viability assay of indicated Caki-1 cells treated with 400 µM TBHP (A) or 12.5 µM Erastin (B). **C-D,** Cell viability was measured in KDM5C-knockdown ACHN cells treated with 200 µM TBHP (C) or 1 µM Erastin (D) combined with or without 20 µM glycogen phosphorylase inhibitor (GPI). **E,** Representative images showing colonies of the KDM5C-knockdown or control ACHN cells on soft agar. **F,** 4-HNE immunohistochemical staining of kidney tissue from wild-type C57 or *kdm5c*^-/-^ mice at indicated month, scale bars, 20 µm. **G,** Representative image of BALB/c Nude mice xenograft tumors derived from Caki-1-shCtrl and Caki-1-shKDM5C cells (left), line chart represented variation of tumor volume(right), (*n* = 6 per group). **H,** Hematoxylin and eosin (H&E), PAS, and 4-HNE immunohistochemical staining of tumor xenografts derived from Caki-1-shCtrl and Caki-1-shKDM5C cells. Scale bars, 20 µm. *P* values were calculated using paired t-test (A-D). Data represent means ± SD.

In contrast, KDM5C depletion conferred the resistance of several ccRCC cell lines to TBHP and Erastin (**Figure 6A and 6B, Figure S6A-D**). Notably, the resistance was readily diminished in the presence of the glycogen phosphorylase inhibitor (GPI) (**Figure 6C and S6D**), suggesting that loss of KDM5C protein mediated glycogen reprogramming was essential for resistance to ROS and ferroptosis. Moreover, KDM5C depletion promoted the anchorage-independent cell growth (**Figure 6E and Figure S6E**).

Ferroptosis has been implicated in age-related disease 20, 21 and recent study revealed that elevated 4-HNE level could be observed in kidney tissue of aging mouse model^22^. Consistently, our result showed that 4-HNE was almost not detected in all the two months old mice but was presented in ten months old mice kidney tissue (**Figure 6F**), implying that the lipid peroxides accumulation and ferroptosis occur during aging. However, the 4-HNE signal became almost undetectable in 10 months *kdm5*^-/-^ mice, reinforcing the idea that KDM5C deficiency suppresses the lipid peroxidation and ferroptosis. Because ferroptosis kills malignant cells and have a tumor-suppressor function 23, our results might explain why transformation of renal cysts into hyperplastic lesions were only observed in *kdm5*^-/-^ mice but not in the normal controls (**Figure 2**).

Furthermore, knocking down KDM5C promoted tumor growth (**Figure 6G**). Consistently, xenograft tumors with KDM5C depletion exhibited an increased glycogen content and decreased 4-HNE level (**Figure 6H**). These observations suggested that KDM5C deficiency elicit ccRCC-specific metabolic phenotypes and confered resistance of ferroptosis.

### Cancer-associated *KDM5C* mutations are defective in inhibiting glycogen accumulation and promoting ferroptosis

**Figure 7:**
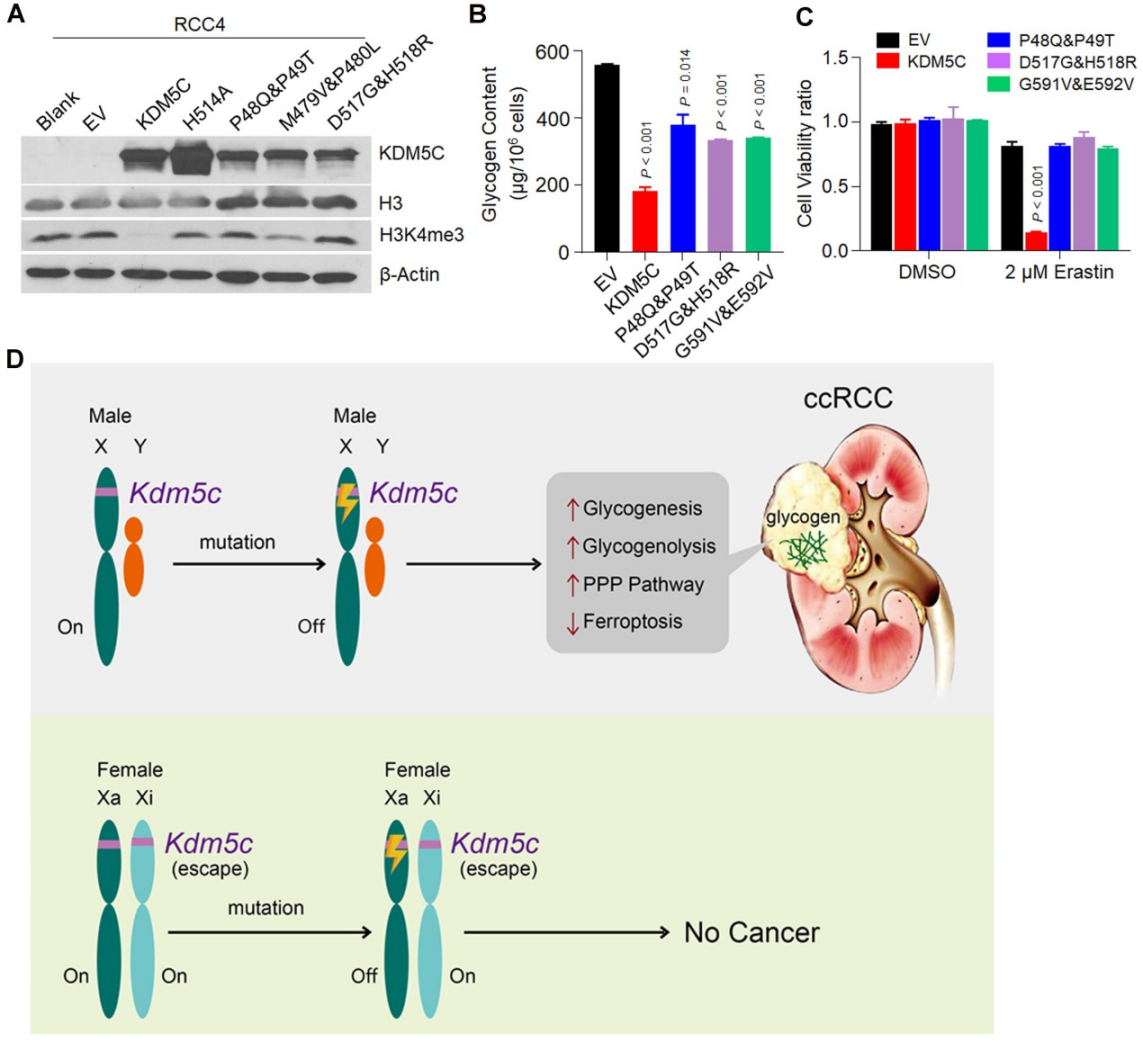
** Clinically associated KDM5C mutants fail to reduce the glycogen level and suppress ferroptosis. A,** Western blot analysis of KDM5C, H3 and H3K4me3 expression levels in indicated RCC4 cell lines. **B,** Bar graph showing the intracellular glycogen level in indicated cells. **C,** Cell viability of indicated cells treated with 2 µM Erastin for 48h. *P* values were calculated by paired t-test (B, C). Data represent means ± SD. **D,** A model of inactive *KDM5C* mutation and ccRCC onset. In females, there are two active alleles of *KDM5C*, and females are therefore protected from complete gene loss after a single alteration. In contrast, in males, one mutation can inactivate the only allele of *KDM5C* gene to elicit metabolic alterations and suppress ferroptosis, and males are therefore more likely to develop ccRCC.

Many *KDM5C* missense and deletion/insertion mutations have been identified in ccRCCs. Importantly, KIRC patients with *KDM5C* mutations had poor prognosis (**Figure S7A**). We found that a large proportion of the mutations were enriched in the JmjN and JmjC domains (**Figure S7B**), thereby affecting the histone demethylase activity of KDM5C. To evaluate the effects of these cancer-related *KDM5C* mutations on glycogen accumulation and ferroptosis, we selected several JmjN and JmjC domains associated-missense mutations and constructed the corresponding RCC4 cell lines that stably expressed the mutant proteins (**Figure 7A**). Interestingly, all of these mutants behaved as H514A, because they failed to reduce the glycogen and G6P levels as efficiently as wild-type KDM5C (**Figure 7B and Figure S7C**), and none of them sensitized cells to ferroptosis or ROS inducer (**Figure 7C and Figure S7D**). Taken together, our results revealed the significance of these cancer-associated *KDM5C* mutants in glycogen laden formation and ferroptosis sensitivity. Collectively, our study suggested that inactive mutations in KDM5C drove the progression of ccRCC by regulating glycogen metabolism-related ferroptosis.

## Discussion

Oncogenic metabolism and epigenetic reprogramming have become central features of ccRCC. Although the male predominance of ccRCC is recognized, its correlation with the characteristics of ccRCC is unclear. In this study, we provided evidence that the deficiency of an epigenetic modifier KDM5C, which was predominantly mutated in male ccRCC patients, contributed to the metabolic phenotypes of ccRCC, such as glycogen laden and PPP up-regulation. Importantly, the type of redox metabolism related to ferroptosis was also significantly altered when KDM5C was depleted. Based on previously published data and the results presented here, we proposed a model for the inactive *KDM5C* mutation in ccRCC. As shown in **Figure 7D**, there are two active alleles of *KDM5C* in females; females are therefore protected from complete gene loss after a single gene alteration. In contrast, in males, one renal cell mutation inactivated the only allele of the *KDM5C* gene, thereby probably promoting tumorigenesis by increasing glycogenesis/glycogenolysis and suppressing ferroptosis. Therefore, males would be more likely to develop ccRCC. Although there is a *KDM5C* homolog *KDM5D* in the Y chromosome, it is worth noting that male ccRCCs with a *KDM5C* loss-of-function mutation were more likely to lose Y chromosome than those without such a mutation 14, indicating the necessity of KDM5C deficiency in ccRCC development. Coincidentally, our recent study showed that high-risk human papillomavirus (HPV) protein degraded the KDM5C protein and promoted cervical cancer progression 24, again reinforcing the idea that KDM5C can play a protective role in the prevention of female malignancies.

Several large-scale clinical genomic profiling has shown the presence of a large number of chromatin modifier genes in ccRCCs, including *PBRM1*,* SETD2*,* BAP1*, *KDM5C*, and *MLL2* 25, 26, but none of them is associated with the characteristic metabolic phenotypes of ccRCC. Oncogenic metabolism is usually caused by oncogene activation (i.e., RAS and MYC), metabolic enzyme alterations (i.e., FBP), or loss of tumor suppressors (i.e., TP53). Our* in vitro* and* in vivo* data indicated that the epigenetic factor KDM5C, as a tumor suppressor, was a novel metabolic regulator, and its absence caused ccRCC-specific metabolic phenotypes, thus providing a direct link between epigenetic abnormalities and cancer metabolism.

KDM5C was initially shown to influence neuronal survival and dendritic development, and its deficiency contributes to the X-linked mental retardation 27. With the rapid development of cancer genomics, *KDM5C* gene mutation has been defined as a common mutation across a number of cancer types 28, including KIRC 29. KDM5C was reported to be a tumor suppressor in ccRCC since knockout of KDM5C in ccRCC promoted the expression of several HIF1α-responsive genes and tumorigenesis 30. Recent study showed that HIF1α was essential for tumor formation and HIF-1α regulates glycolysis 31, our data further supported the notion that KDM5C partially inhibited the elevated expression of HIF1α- and metabolism-related genes, which may be caused by loss of VHL. Moreover, as an epigenetic modifier, KDM5C regulated the expression of HIF1α-independent genes, including *G6PD*. Therefore, the deletion of KDM5C in VHL null cells precisely coordinated the glycogenesis/glycogenolysis and subsequent PPP to ensure efficient glucose flux through PPP. This conclusion could be further validated by the evidence presented in this study that an intrinsic frame-shift *KDM5C* mutation was identified in the glycogen-enriched and VHL^-/-^ ccRCC cell line, RCC4 (**Figure 1**). Previous study indicated that VHL re-expression in RCC10 cells reduced NADPH 5, which was not found in RCC4 cells in our study, we speculated that because fructose-1,6-bisphosphatase 1 (FBP1) coordinates with HIF proteins and mediated effects on glucose metabolism, and fructose-1,6-bisphosphatase 1 (FBP1) is almost absent in RCC4 cells but expressed in RCC10 cells 5, which might help to the explain the different effect of VHL on PPP pathway in two lines. Moreover, up-regulated PPP and inactive *KDM5C* mutation were associated with poor prognosis in ccRCC patients, our study therefore provided mechanistic insight into how inactive *KDM5C* mutations strengthened PPP and became a high-risk factor.

Glycogen metabolism has become a well-recognized feature of cancer cells since it is up-regulated in many tumor types to maintain cell proliferation 32 and promote cell metastasis through glycolysis 33, however, its exact role in ccRCC is not fully understood. A recent study showed that induced glycogen accumulation could be directed to PPP in T memory cells 7. Our study showed that abnormal epigenetic modification was sufficient to simultaneously promote glycogen accumulation, degradation, and subsequent PPP, thereby significantly increasing the NADPH level, enhancing the anti-ferroptosis activity, and displaying a distinct aspect of glycogen function during tumorigenesis.

One of the key challenges in cancer treatment is how to effectively kill cancer cells while keeping healthy cells unaffected. CcRCC has been defined as resistant to conventional chemotherapy and radiotherapy, therefore exploiting other forms of non-apoptotic cell death may help to open new therapeutic avenues for eliminating ccRCC cells. Ferroptosis is defined as iron-catalyzed necrosis due to excessive peroxidation of polyunsaturated fatty acids (PUFAs) and is closely related to NADAH 34, 35, GSH, and other factors 19. Ferroptosis can be epigenetically regulated. For example, deubiquitinating enzyme BAP1 can reduce histone 2A ubiquitination (H2Aub), thereby inhibiting *SLC7A11* expression and promoting ferroptosis 36. Our results revealed a new epigenetic regulation pattern of ferroptosis, namely, the inactive *KDM5C* mutation in ccRCC suppressed the ferroptosis by increasing the H3K4me3 levels on the promoters of several glucose metabolism-related genes (**Figure 3C-3G**), which resulted in altered glycogen metabolism, as well as elevated NADPH and GSH. Meanwhile, the presence of glycogenolysis inhibitor GPI efficiently re-sensitized KDM5C-depleted cells to the ferroptosis inducer (**Figure 5** and **Figure 6**), indicating that targeting the glycogen metabolism to induce ferroptosis in KDM5C-deficient ccRCCs might offer intriguing therapeutic interventions.

In summary, we demonstrated that the inactive mutation of X-inactivation escaping gene *KDM5C* promoted glycogen reprogramming and subsequent PPP, and induced the ferroptosis resistance, thereby expanding the repertoire of KDM5C functions that were essential for tumorigenesis and male predominance of ccRCC.

## Materials and Methods

### Vectors and plasmids

KDM5C and VHL were subcloned into pHAGE for mammalian cell expression. A series of KDM5C mutations were generated by site-directed mutagenesis PCR from the pHAGE-KDM5C plasmid. shRNAs targeting human KDM5C and/or VHL were constructed using pLKO.1. The targeted sequences are as followed: shCtrl: 5'CAACAAGATGAAGAGCACCAA-3'; shKDM5C-1: 5'-AGTACCTGCGGTATCGG- -TATA-3'; shKDM5C-2: 5'-GCCACACTTGAGGCCATAATC-3'; shVHL-1: 5'-TATC- -ACACTGCCAGTGTATAC-3'; shVHL-2: 5'-CCATCTCTCAATGTTGACGGA-3'. All the constructs were validated by DNA sequencing.

### Cell culture

The human ccRCC cell lines (RCC4, ACHN, 786-O, 769-P, Caki-1 and A498) and human renal epithelial cell (HK-2) were obtained from ATCC and were grown in high-glucose Dulbecco's modified Eagle's medium (DMEM, Hyclon) supplemented with 10% fetal bovine serum (FBS, AusgeneX) in a humidified atmosphere containing 5% CO_2_ at 37 °C.

To generate cell lines stably overexpressing wild type or mutant KDM5C and VHL, or stably knocking down KDM5C and/or VHL, HEK293T cells were transfected with appropriate constructs and two packaging plasmids psPAX2 and pMD2.G using Lipofectamine 2000 reagent (Invitrogen) according to the manufacturer's instructions. 60 h later, lentivirus particles in the medium were collected and filtered, and then were added to target cells along with 8 ug/mL polybrene. Infected cells were then selected in puromycin-containing medium. Protein levels of target genes were determined by western blot with corresponding antibodies.

### RNA extraction and qRT-PCR

Total RNA was extracted using TRIzol (Invitrogen, 15596018) according to the manufacturer's instructions. cDNA was then obtained using All-in-One cDNA synthesis SuperMix (Bimake, B24403). RT-PCR was performed with SYBR Green Real time PCR Master Mix (Bimake, B21802) on CFX Connect Real Time PCR Detection System (Bio-Rad). β-Actin or GAPDH expression was used for normalization. For quantification of gene expression, the 2^-∆∆Ct^ method was used. Each reaction was done in triplicates. Primer sequences are shown in Table S1.

### Western Blotting

Cells were lysed in RIPA buffer (Beyotime, P0013K) containing 1X protease inhibitor mixture (MCE, HY-K0010). Total protein concentrations were measured by BCA kit (Beyotime, P0012). An amount of 20-100 μg of proteins were separated on 8-15% sodium dodecyl sulphate-polyacrylamide gel electrophoresis (SDS-PAGE) and transferred to nitrocellulose membrane. Blots were then blocked and incubated with primary antibodies overnight at 4 °C. Then appropriate horseradish peroxidase (HRP)-conjugated secondary antibodies were incubated with the blots for 1 hour at room temperature. The protein bands were visualized using ECL and exposured to X-ray film. The primary antibodies used are KDM5C (Bethyl, A301-034A), VHL (ABclonal, A0377), H3K4me3 (ABclonal, A2357), H3 (CST, 2650), α-Tubulin (Proteintech, 66031).

### RNA-seq

Total RNA was extracted in triplicate from RCC4-PHAGE (EV) and RCC4-KDM5C cells, or ACHN-shCtrl and ACHN-shKDM5C cells using TRIzol (Invitrogen, 15596018). Purity of RNA samples was determined with NanoPhotometer (IMPLEN), concentration was measured by Qubit 3.0 fluorometer (Life Technologies) and the sample integrity was evaluated using Agilent 2100 RNA Nano 6000 Assay Kit (Agilent Technologies). The RNA-seq were performed by Beijing Novogene Biotech Co. Ltd. Briefly, sequencing libraries were constructed using the NEBNext Ultra RNA Library Prep Kit for Illumina (NEB, USA). Clustered sequences were generated using TruSeq PE Cluster Kit v3-cBot-HS (Illumina). For the data analysis, index of the reference genome was built using Hisat2 v2.0.5 and paired-end clean reads were aligned to the reference genome using Hisat2 v2.0.5. FeatureCounts v1.5.0-p3 was used to count the reads numbers mapped to each gene. Differential expression analysis of two groups was performed using the DESeq2 R package (1.16.1). To obtain the Kyoto Encyclopedia of Genes and Genomes (KEGG) result, the ClusterProfiler R package softeware was employed to analyze the enrichment of differentially expressed genes.

### Whole exome sequencing

RCC4 and HK2 cells were grown in 100 mm dishes until 80% confluency. Genomic DNA were extracted with TIANamp Genomic DNA Kit (Tiangen Biotech, DP304-03). Using agarose gel electrophoresis to analyze the integrity and purity of DNA, and the concentration was determined by Qubit 2.0. The exome sequencing were performed by Beijing Novogene Biotech Co., Ltd. In brief, DNA library were constructed using Agilent SureSelect Human All Exon V5/V6 Kit, then the quality was assessed using Q-PCR. The sequencing was performed with Illumina HiSeq PE150. Raw data were processed and aligned to human reference genome (GRCh37.p13) through BWA and Samblaster software. The mutations were analyzed by SAMtools and ANNOVAR software.

### Periodic Acid-Schiff staining (PAS)

Glycogen was detected with Periodic Acid-Schiff staining kit (Leagene, DG0011) according to the manufacturer's instructions. Briefly, cells were seeded to a 6 wells plate at 60% confluence, the medium was removed and the cells were washed twice with PBS, fixed with 4% paraformaldehyde for 20 min, followed by washing it with water. The cells were then incubated with periodic acid solution for 20 min and washed with deionized water. Then the cells were stained with Schiff reagent for 25 min and rinsed with tap water. For visualizing cell nuclear, the cells were counterstained using Mayer's hematoxylin for 3 min, the stained cells were observed by a microscope (Olympus, IX53+DP80).

### Chromatin immunoprecipitation-PCR (ChIP-PCR)

Cells were grown in 100 mm dish until 90% confluency and fixed with 1% formaldehyde solution for 15 minutes. The reaction was quenched with 0.125 M glycine for 10 minutes before washing the cells with ice-cold PBS. Cells were scraped off and resuspended in Lysis Buffer (10 mM Tris-HCl pH 8.0, 100 mM NaCl, 1 mM EDTA, 0.5 mM EGTA, 0.1% Na-Deoxycholate, 0.5% N-lauroylsarcosine (N-dodecane Sodium sarcosinate)), then sonicated in ice-water bath to obtain DNA fragments in the range of 200-500bp. Clarified lysates were diluted with ChIP dilution buffer ( 1% Triton X-100, 1 mM EDTA pH 8.0, 50 mM Tris-HCl pH 8.0, 150 mM NaCl, 0.1% Na-Deoxycholate and protease inhibitor cocktail), and incubated overnight at 4 °C with appropriate primary antibodies. rProtein A/G Beads 4FF (Smart-lifesciences, SA032200) were blocked with salmon sperm DNA (Sigma, D7656) for 2 h at 4 ℃. Then the Protein-DNA complex incubate with blocked Protein A/G beads at 4 °C for 4 h. Beads were washed once with each of the following buffers : low salt wash buffer (0.1% SDS, 1% Triton X-100, 1 mM EDTA, 25 mM Tris-HCl pH 8.0, 150 mM NaCl, 0.1% Na-Deoxycholate), high salt wash buffer (0.1% SDS, 1% Triton X-100, 1 mM EDTA, 25 mM Tris-HCl pH 8.1, 500 mM NaCl, 0.1% Na-Deoxycholate), LiCl wash buffer (0.5 M LiCl, 0.7% Na-Deoxycholate, 1 mM EDTA, 10 mM Tris-HCl pH 8.0, 1% NP-40) and TE buffer (10 mM Tris-HCl pH 8.0, 1 mM EDTA). Complexes were eluted from the beads using Elution Buffer (1% SDS, 0.1 M NaHCO3) at room temperature and cross-links were reversed by the addition of 5 M NaCl solution at 65 °C overnight. ChIP DNA were recovered with Universal DNA Purification Kit (Tiangen Biotech, DP214). qRT-PCR were performed on the CFX Connect Real-Time PCR Detection System (Bio-Rad) using SYBR Green qPCR Master Mix. ChIP-PCR primer sequences used in this assay were listed in Table S2.

### Glycogen measurement

Glycogen levels were measured using the Glycogen Colorimetric Assay Kit Ⅱ (Biovision, K648) following the manufacturer's instructions. Briefly, cells (2X10^5^) were homogenized with 400 μl ddH_2_O on ice and then boiled for 10 minutes. Then spun and collect supernatants to be assayed for glycogen content. Results were normalized by protein concentration or cell number.

### Determination of Glucose-6-Phosphate level

G6P levels were measured using Glucose-6-Phosphate Colorimetric Assay Kit (Biovision, K657) following the manufacturer's instruction. Cells (2X10^6^) were homogenized in 400 μl ice cold PBS, centrifuged to remove the insoluble materials, then deproteinized using a 10 KDa Centrifugal Filter Unit with Ultracel-10 membrane (Millipore, MRCPRT010), and the filtrate was used for the G6P level determination.

### NADP/NADPH Quantification

NADP/NADPH ratio were determined with the NADP/NADPH Quantification Colorimetric Kit (BioVision, K347), according to the manufacturer's instruction. In brief, cells (4×10^6^) were lysed with 800 μl extraction buffer on ice, spun and the supernatant was divided into two aliquots, one was directly used for determination of total NADP^+^ and NADPH, another one was heated at 60 °C for 30 min and used to detect NADPH only. Then each of the samples mixed with the reaction buffer were incubated at room temperature for 1-3 h. Datas were obtained by using a spectrophotometer at the absorbance of 450 nm.

### GSH assay

Glutathione (GSH/GSSG/Total) Fluorometric Assay Kit (Biovision K264) was used to detect the intracellular reduced glutathione levels according to the manufacturer's instructions. Cells (2×10^6^) were seeded on a 100 mm dish and cultured until 90% confluency in the routine culture medium. Cells were trypsinized and washed three times with ice cold PBS, extracted with ice cold Glutathione Assay Buffer, then added PCA and vortexed several seconds on ice, and collected the supernatant, KOH was used to neutralize the sample before it was diluted with assay buffer. Incubating samples with OPA Probe at room temperature for 40 minutes and read samples on a fluorescence plate reader (Spectramax i3x, Molecular Devicesmd).

### ROS Detection

ROS levels were measured using CellROX Green Flow Cytometry Assay Kit (Invitrogen, C10492) following manufacturer's instructions. Cells (2×10^6^) were grown in 100 mm dishes overnight and then treated with or without 200 μM TBHP for 2 hours, negative controls were induced using N-acetylcysteine (NAC) for 2 hours in advance. Then harvested the cells and added the CellROX reagent at a final concentration of 500 nM to the samples for 30 min, washed sample once with ice-cold PBS and incubated with 5 mM SYTOX® Red Dead Cell stain solution for 30 min at room temperature. Result were obtained by using a flow cytometer (LSRFortessaX20, BD).

### Lipid peroxidation assay

Cells (2×10^6^) were grown in 100 mm dishes and then treated with 2.5 μm Erastin or DMSO for 6 hours. Cells were then trypsinized and washed, BODIPY 581/591 C11 (Invitrogen) was added for 30 min, and stained with SYTOX Dead Cell Stains (Invitrogen) for 30 min before transferred to FACS tubes in PBS. Flow cytometry was performed using the LSRFortessaX20 (BD).

### Cell survival assay

The cell viability was measured using the CCK8 kit (MCE, HY-K0301) in the 96-well plate. The cells were seeded at 30-50% confluence (depends on the cell's growth rate), when the cells attached, firstly incubated with Ferrostatin-1 (Ferr-1) (Sigma, SML0583), Z-VAD-FMK (Z-VAD) (Selleck, S7023), Necrostain-1s(Nec-1s) (Selleck, S8641), Deferoxamine mesylate (DFO) (Selleck, S5742) or glycogen phosphorylase inhibitor (GPI) (Merck, 361515) for 12 h. Then co-treated with Erastin (Sigma, E7781) or tert-Butyl hydroperoxide (TBHP) (Sigma, 458139) for indicated time. Then all medium was removed, replaced with the fresh medium containing CCK8 reagent and incubated for 2-4 h, the plates were read by microplate reader at 450 nm.

### Electron Microscopy

Tissue (≤1 mm^3^) were fixed overnight in 2.5% glutaraldehyde in 0.1 M phosphate buffer, rinsed using PBS and then post-fixed in 1% osmium tetroxide buffer at room temperature for 2 hours, then rinsed 3 times with PBS, dehydrated in various concentrations of ethanol (50%, 75%, 80%, 95%, 100%) for 15 minutes each and then washed twice with acetone. The samples were embedded in embedding media and cured for 9 h at a 70 °C vacuum oven and were sliced. Ultrathin sections of 50-70 nm width were fixed onto 200 mesh copper grids, stained with 2% uranyl acetate and lead citrate before being scanned by transmission (frozen) electron microscopy (Tecnai G20 TWIN).

### Soft agar assay

The soft agar was prepared in 6-well plates and consist with two layers. Bottom layer contains 0.5 % agar. To prepare top layer, cells were collected and pipetted to single-cell suspension with complete culture medium. Each cell suspension (1×10^4^ cells) mixed with 1.4% agarose at ratio of 3:1 in volume (the final concentration of agarose was 0.35%) and were poured onto the bottom layer. The resulting soft agar were then covered with appropriate medium (with or without inhibitors), changing medium each other day for four weeks (ACHN) or eight weeks (RCC4, A498, Caki-1). The formed colonies were stained with 0.01% crystal violet in PBS.

### Xenograft assay

For the xenograft of RCC4 cells, four-week-old female NOD.CB1 - Prkdcscid/NcrCrl mice were acquired from Beijing Vital River Laboratory Animal Technology (China) and were housed under specific pathogen-free conditions in Animal Experiment Center-Animal Biosafety Level-III Laboratory of Wuhan University. The animals were fed with standard laboratory mice diet and water ad libitum. At 6th week, mice were injected with 100 μL of stable RCC4-EV or RCC4-KDM5C cells suspended in Matrigel Basement Membrane Matrix (Corning, 356234) at a population of 1 × 10^7^ cells into the left or right dorsal flank subcutaneously after alcohol sterilization of injection site skin surface. On day 7 after injection, the mice were randomly divided into 2 groups and treated with Liproxstatin-1 (10 mg/kg, MCE HY-12726) or vehicle control (1% DMSO in PBS) each other day by i.p. injection.

For Caki-1, four-week-old male BALB/c nude mice (Beijing HFK Bioscience (China)) were used. At 6th week, Caki-1-shCtrl or Caki-1-shKDM5C cells were randomly injected into the left or right dorsal flank, 4 × 10^6^ cells per needle.

Tumor size of each mice were measured each other day using digital vernier caliper. Tumor volume was calculated with the formula: tumor volume (mm^3^) =length (mm) × width (mm^2^) × 0.5. All animal experiments performed were approved by and conform to the guidelines of Animal Research Ethics Board of Wuhan University.

### Generation of *KDM5C* knockout mice

*KDM5C* knockout C57BL/6 mice was generated by CRISPR/Cas9-mediated genome engineering (Cyagen Biosciences Inc. China). The gRNAs designed to target specific sites of KDM5C in the mouse genome (GenBank: NM_013668.4) shown in Figure S6. We used two distinct gRNAs and they were co-injected with Cas9 to result in knockout pups with critical region deletion. Ten days after the mouse born, cut off the toes of the mouse and extracted the DNA with TIANamp Genomic DNA Kit (Tiangen Biotech, DP304-03). The efficiency of gene knocked out was evaluated by PCR analysis using specific primers designed according to the depleted region. The verified positive mice were retained to continue breeding the next generation.

- mKDM5C-gRNA1: GATGGGGAGTAGCCCTATCCTGG;
- mKDM5C-gRNA2: GGGATACTTTAGGCTACCTTAGG.

### Heavy isotope tracer analysis

RCC4-vector or RCC4-KDM5C cells were seeded in the 100 mm dishes. After cells were completely adhered, replaced original medium with fresh medium containing equal amount of 1,2-^13^C_2_-glucose and ^12^C-glucose and cultured for 24 h, cells were harvested for measuring the ^13^C carbon tracing and intracellular metabolic flux, which were largely performed in Biotree Co Ltd. Briefly, the samples and standards were measured using a LC-MS analysis, which was performed by UHPLC system coupled with a QTOF mass spectrometer (AB 6600 TripleTOF, SCIEX, Canada) in negative mode, respectively. All samples were randomly injected during data acquisition. Blank sample (100% acetonitrile) and QC sample were injected every 8 samples to monitor the stability of LC-MS. A Waters a HILIC HPLC carbon column (Amide 4.6×100 mm ID 3.5 μm; Part No: 186004868, Waters). Mobile phase buffer A 1 L contained 20 mM ammonium hydroxide, 20 mM ammonium acetate, 95% water and 5% (v/v) acetonitrile, pH 9.0 at 25 °C. While mobile phase buffer B was 100% acetonitrile. The column was maintained at a controlled temperature of 30 °C and was equilibrated with 15% buffer A for 3 min at a constant flow rate of 350 μL/min. Aliquots of 15 μL of each sample were loaded onto the column and compounds were eluted from the column with a linear gradient of 15-50% buffer A from 4^th^ to 16^th^ min, and then increased to 85% buffer A from 16^th^ min to 20^th^ min and decreased to 15% buffer A from 21th to 23^rd^ min, and the column was washed for a 2 min with 15% buffer A. The ion transfer tube temperature was set to 350 °C and vaporizer temperature was 270 °C. The instrument was run in negative mode with a spray voltage of 3,000, sheath gas 40 and Aux gas 5.0.

For intracellular metabolic flux, all samples and standards were measured using a TSQ VANTAGE interfaced with Ultimate 3000 Liquid Chromatography system (Thermo Scientific), equipped with a HILIC HPLC carbon column (Amide 4.6×100 mm ID 3.5 μm; Part No:186004868, Waters). Mobile phase buffer A was 1L contained 20 mM ammonium hydroxide, 20 mM ammonium acetate, 95% water and 5% (v/v) acetonitrile, pH 9.0 at 25 °C. While mobile phase buffer B was 100 % acetonitrile. The column was maintained at a controlled temperature of 30 °C and was equilibrated with 15% buffer A for 3 min at a constant flow rate of 350 μl/min. Aliquots of 10 μl of each sample were loaded onto the column and compounds were eluted from the column with a linear gradient of 15-50% buffer A from 4th to 16th min, and then increased to 85% buffer A from 16th min to 20th min and decreased to 15% buffer A from 21th to 23th min, and the column was washed for a 2 min with 15% Buffer A. The ion transfer tube temperature was set to 350 °C and vaporizer temperature was 270 °C. The instrument was run in negative mode with a spray voltage of 3,000, sheath gas 40 and Aux gas 5.0. The 7-8 concentrations (from low to high) of the different standard mix were measured using multiple reactions monitoring mode (MRM) with optimal collision energies to produce standard curve.

### Tissue microarray IHC analysis

Tissue chips of renal clear cell carcinoma were obtained from Shanghai Outdo Biotechnology (HKid-CRCC060PG-01). It contains 30 paired tumor tissues and corresponding adjacent normal tissues. For Immunohistochemistry, Firstly, slides were immersed in xylene for 10 minutes and rehydrated, rinsed with distilled water. Then the slides covered with citrate Buffer, heated in the microwave for 10 minutes. When cooled down, rinsed three times with TBST for 3 minutes each, incubated with 1% H_2_O_2_ for 10 minutes and then rinsed, blocking the chips with 5% BSA in TBST for 1 h, and then incubated with primary antibody (Ki67, Proteintech 27309; KDM5C, Origene AM50089PU-N; 4-HNE, Abcam ab46545) in TBST overnight at 4 °C. After removing the unbound the primary antibody, chips were incubated with peroxidase labeled polymer for 30 minutes, rinsed as above, applied the substrate (mixed one drop of liquid DAB plus chromogen with 1ml substrate solution immediately) to the slides and incubated for 5-10 minutes till brown color develops, then rinsed gently with distilled water. To stain the nuclear, immersed in hematoxylin for 3-5 minutes, then rinsed with distilled water. Finally, immersing slides sequentially into 60%, 80%, 95%, 100% ethanol for 5 minutes each, then in xylene for 5 minutes, covered the chip with a cover slip, air-dried in a fume hood. The images were obtained by using Pannoramic MIDI Digital Scanner (DHISTECH, Hungary).

### Statistical analysis

Statistical analysis (two-tailed Student's t-test) was performed using GraphPad Prism. For box plots, the upper and lower edges of the box indicate the first or third quartiles of the data, and the middle line indicates the median. The top and bottom edges of the whisker indicate the maximum and minimum of the data. The fold changes of altered gene expression in KDM5C knock down cells were derived from the data of RNA-Seq, and the *P*-value was calculated by t-test and adjusted using the False Discovery Rate (FDR) method in Fig.3A. For KEGG enrichment analysis in Fig.3B, the *P* value was determined by Fisher exact test. For survival analysis of patients with *KDM5C* gene mutation in KIRC, the *P* value was obtained by Gehan-Breslow-Wilcoxon test. All the *P* value calculated above are displayed in the corresponding figures. Each experiment was repeated independently more than two times with similar results.

Clinical data were downloaded from UCSC Xena (http://xena.ucsc.edu/welcome-to-ucsc-xena/) and cBioPortal (https://www.cbioportal.org/) and were analyzed using the Prism. For the gender percentage of several mutated genes in Fig.1G, the sample of gene mutation patients in TCGA Kidney Clear Cell Carcinoma (KIRC) derived from cBioPortal and the gender information of mutated patients obtained from UCSC Xena. For the indicated gene expression level in tumor and adjacent normal tissues in KIRC in Fig.S1D and S3D-H, the data of gene expression from RNAseq HTSeq-FPKM of TCGA KIRC was downloaded using UCSC Xena. For survival analysis of the patients with *KDM5C* gene mutation in KIRC in Fig. S7A, we used cBioPortal to analyze the prognosis.

## Supplementary Material

Supplementary figures and tables.

## Figures and Tables

**Table 1:** A number of genomic alterations were identified in RCC4 cells through comparing the exome sequencing results of HK2 and RCC4 cells

Gene	Mutation
KDM5C	1 frameshift deletion
VHL	1 missense
USP34	1 splicing,1synonymous
TBP	1 nonframeshift deletion,3 synonymous
FOXE1	1 nonframeshift deletion, 2 synonymous
EP400	1 nonframeshift insertion, 1 missense
POLG	1 nonframeshift deletion
CHD2	1 frameshift deletion, 2 synonymous

## Abbreviations

KDM5C: lysine (K)-specific demethylase 5C
ccRCC: Clear cell renal cell carcinoma
VHL: Von Hippel-Lindau
PPP: pentose phosphate pathway
G6PD: Glucose-6- phosphate Dehydrogenase
G6P: glucose-6-phosphate
NADPH: nicotinamide adenine dinucleotide phosphate hydride
GSH: glutathione, reduced form
ROS: reactive oxygen species
PGM1: phosphoglucomutase 1
GYS: glycogen synthase
PPP1R3: protein phosphatase 1 regulatory 3
GP: glycogen phosphorylase
PBRM1: polybromo 1
SETD2: SET domain containing 2
BAP1: BRCA1 (breast cancer 1 ) associated protein 1
PAS: Periodic Acid-Schiff
KIRC: Kidney Renal Clear Cell Carcinoma
IHC: immunohistochemistry
HK2: hexokinase 2
TSS: transcription start site
R5P: ribulose‐5‐phosphate
6-PG: 6‐phosphogluconate
F6P: fructose‐6‐phosphate
TBHP: tert-butyl hydroperoxide
Ferr-1: Ferrostatin-1
DFO: iron chelator deferoxamine
Z-VAD: apoptosis inhibitor Z-VAD-FMK
Nec-1s: necroptosis inhibitor necrostatin-1s
Lip-1: ferroptosis inhibitor liproxstatin-1
EM: electron microscopy
GPI: glycogen phosphorylase inhibitor
4-HNE: 4-hydroxynonenal
