# Supplementary material for: Deficiency of the X-inactivation escaping gene KDM5C in clear cell renal cell carcinoma promotes tumorigenicity by reprogramming glycogen metabolism and inhibiting ferroptosis
Source: Theranostics. 2021 Aug 4;11(18):8674–91. doi: 10.7150/thno.60233 (PMC8419058; doi:10.7150/thno.60233)
Supplement: Supplementary file 1 — Supplementary figures and tables. [file thnov11p8674s1.pdf]

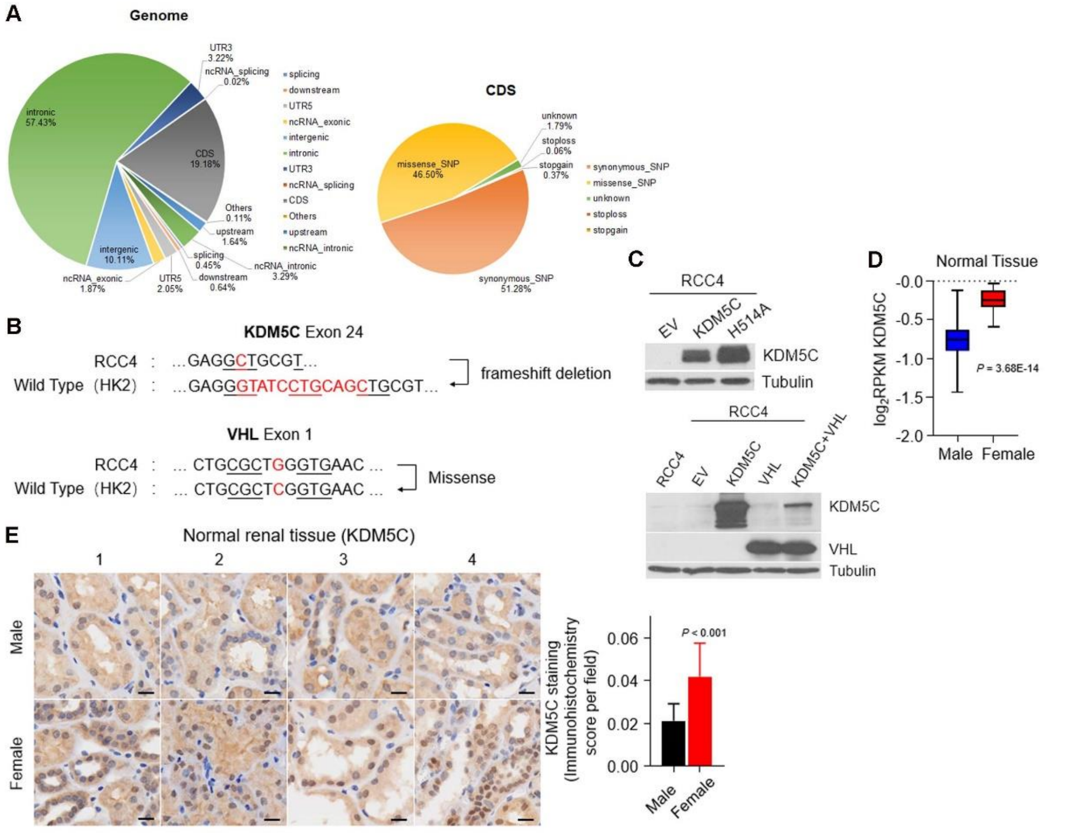

Figure S1: Whole exome sequencing revealed that KDM5C contains a frame-shift mutation in ccRCC cell line with the highest glycogen level

A, The distribution of RCC4 of genomic alterations. Insertion and deletion (INDEL) in the whole genome(left) or coding regions (right).

B, The nucleotide sequences of KDM5C or VHL gene mutated site revealed by the exome sequencing, respectively.

C, The KDM5C and VHL protein level of indicated cells were analyzed by Western Blot.

D, Box plots showing a comparison of KDM5C mRNA level of male and female in normal renal tissues. n=72 (male=52; female=20).

E, KDM5C IHC staining of normal renal tissues. Scale bars, 40 μm.

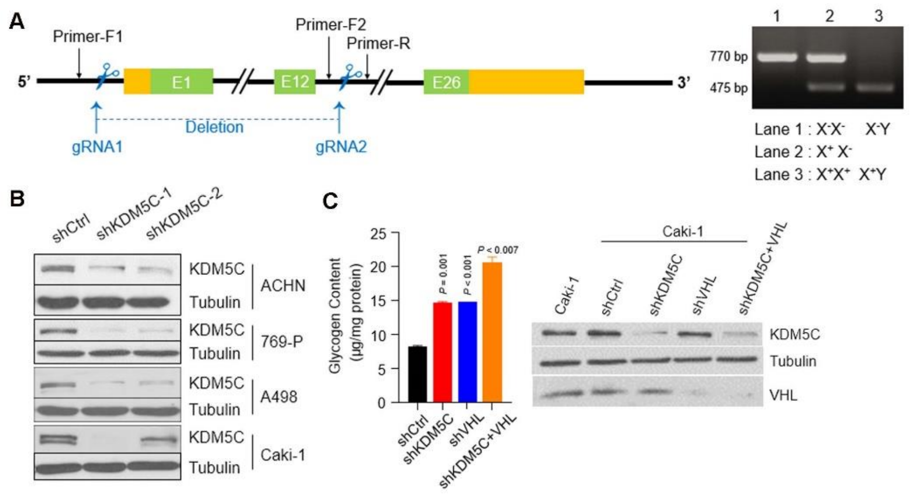

Figure S2: Construction of *KDM5C* knock out mice and stable KDM5C knockdown in multiple ccRCC cell lines.

A, Schematic illustration of the construction of KDM5C knock out mice and the PCR validation of the *Kdm5c*$^{-/-}$ mice.

B, KDM5C knocking down in indicated cells were confirmed by western blotting analysis.

C, The glycogen level of constructed caki-1 cell lines which confirmed by WB.

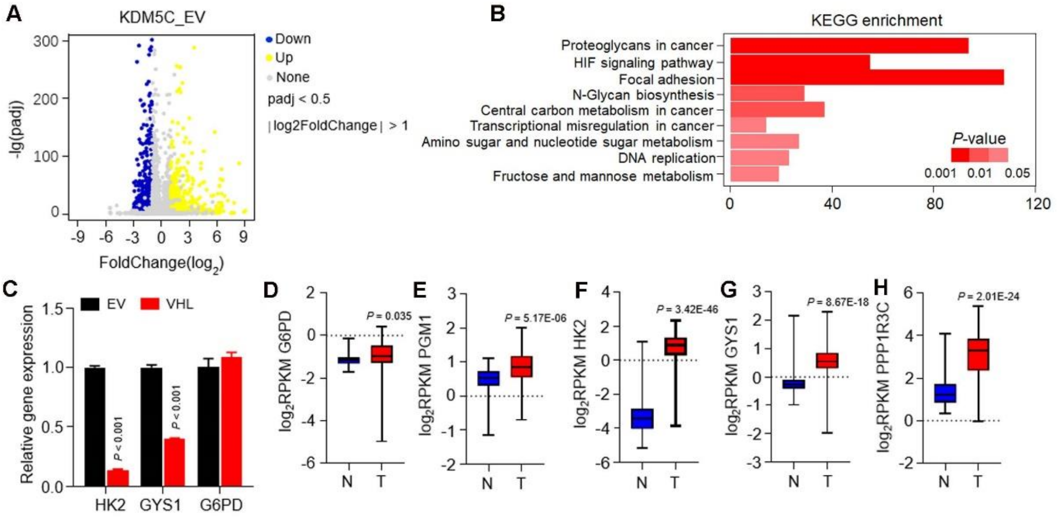

Figure S3: KDM5C and VHL regulates both similar and distinct genes expression

A, Volcano plots of RNASeq data for RCC4 cells transfected with KDM5C or control. Blue, down-regulated genes; yellow, up-regulated genes. The data of fold changes were derived from indicated RNA-Seq, and the *P*-value was calculated by t-test and adjusted using the False Discovery Rate (FDR) method.

B, Kyoto Encyclopedia of Genes and Genomes (KEGG) pathway enrichment analysis of the identified differentially expressed genes (DEGs) from the indicated RNASeq data. *P* -value was determined by Fisher exact test.

C, qPCR analysis for the expression of the indicated genes in the RCC4-EV and RCC4-VHL cells. Each three repeats. *P* values were calculated by paired t-test. Data represent means ± SD.

D-H, Box plots showing a comparison of G6PD(D), PGM1(E), HK2(F), GYS1(G) and PPP1R3C(H) expression levels in normal tissues(N) and corresponding tumor(T) samples. KIRC, n=72 independent samples; adjacent normal tissues, n=72. The upper and lower edges of the box indicate the first or third quartiles of the data, and the middle line indicates the median. The top and bottom edges of the whisker indicate the maximum and minimum of the data. *P* values were calculated by paired t-test. Data represent means ± SD.

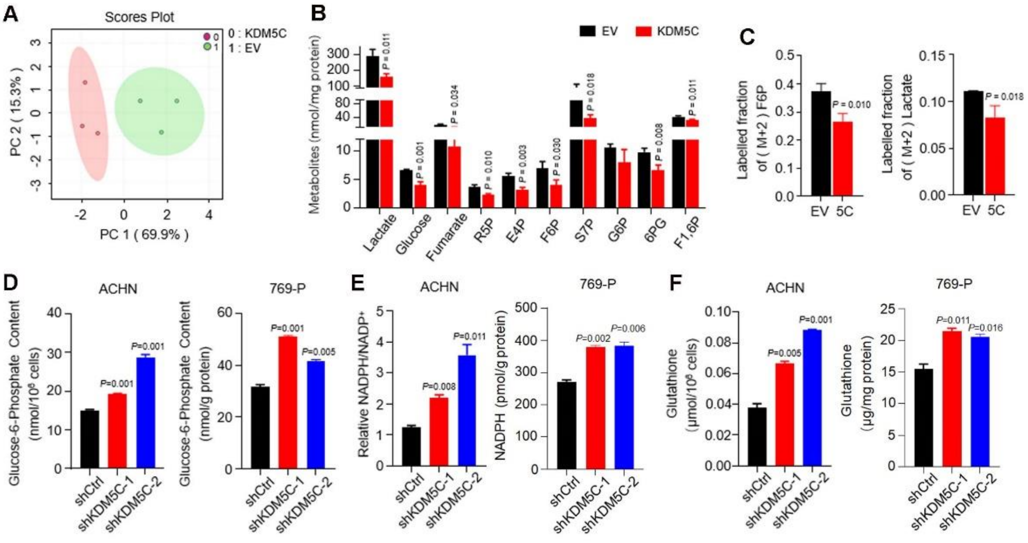

Figure S4: Heavy isotope tracer analysis revealed that KDM5C suppress the flux of glucose to the PPP

A, The Principal Component Analysis (PCA) of the absolute intracellular metabolite data.

B, Bar graph showing a comparison of the indicated metabolites level between RCC4-EV and RCC4-KDM5C.

C, Bar graph showing the changes of (M)+2 Lactate and (M)+2 F6P in the presence of ectopic KDM5C.

D-F, Quantification of glucose-6-phosphate (D), NADPH (E) and glutathione (F) levels of indicated cells. *P* values were calculated by paired t-test. Data represent means ± SD. Data are representative of three independent experiments.

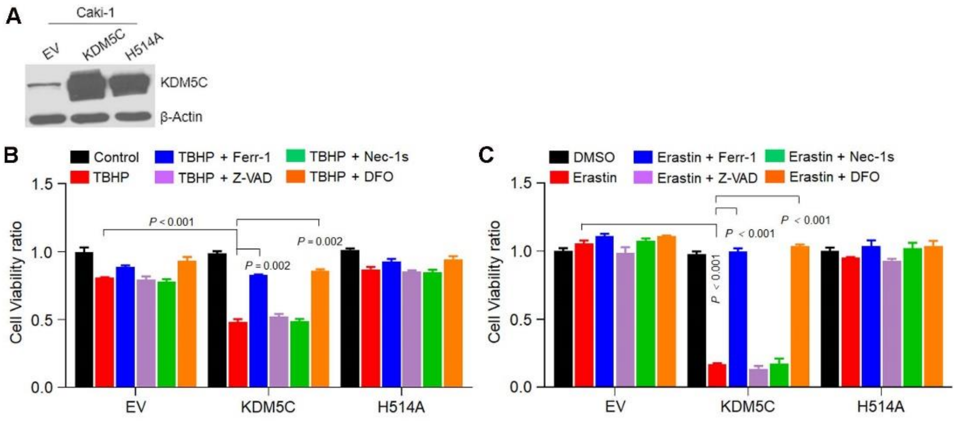

Figure S5: Ectopic KDM5C sensitize caki-1 cells to ferroptosis inducer

A, KDM5C expression level of indicated cells measured by Western Blot.

B, Cell viability of indicated Caki-1 cell lines were treated with 150 μM TBHP combined with 5 μM Z-VAD, 2 μM Nec-1s, 2 μM Ferr-1 or 20 μM DFO for 24 h.

C, Cell viability of indicated Caki-1 cell lines with treated with 2.5 μM Erastin combined with 5 μM Z-VAD, 2 μM Nec-1s, 2 μM Ferr-1 or 20 μM DFO for 24 h.

P values were calculated by paired t-test. Data represent means ± SD. Data are representative of three independent experiments.

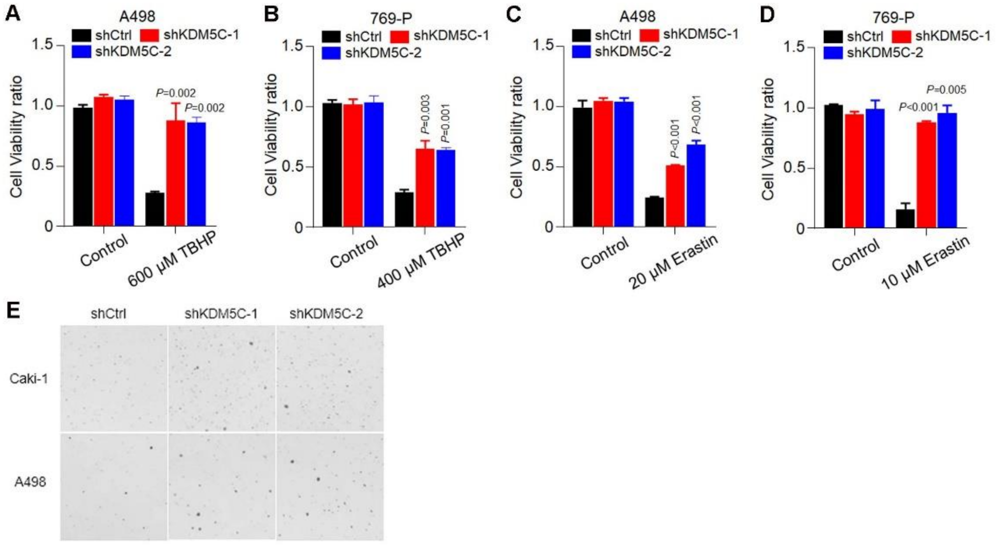

Figure S6: KDM5C depletion confer multiple ccRCC cell lines resistance to ferroptosis
A-B, Cell viability of A498 (A) and 769-P (B) treated with indicated concentration TBHP.
C-D, Cell viability of A498 (C) and 769-P (D) treated with indicated concentration Erastin. *P* values were calculated by paired t-test. Data represent means ± SD.
E, Colony formation of Caki-1 and A498 shKDM5C cell lines.

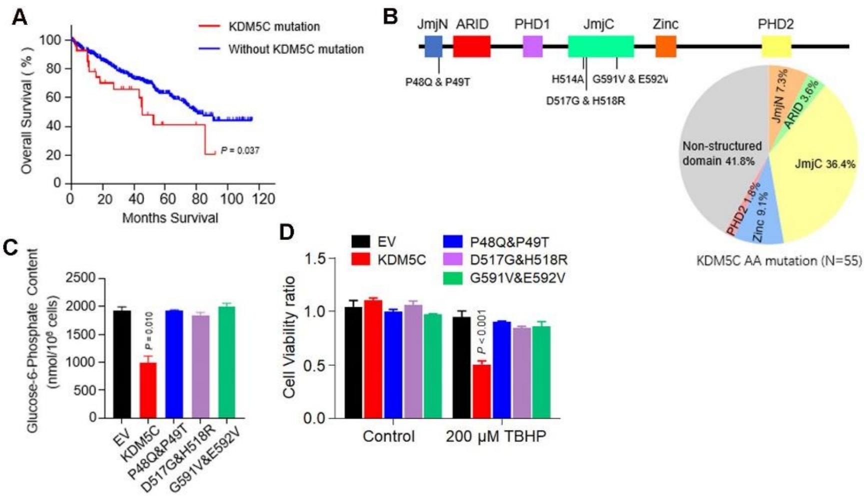

Figure S7: A portion of clinically relevant KDM5C mutants affect glycogen metabolism and ferroptosis

A, Kaplan-Meier plots of KIRC patients stratified by the presence of KDM5C mutations. *P*-value was calculated by the log-rank test.

B, Schematic diagram of missense mutation detected in each of the KDM5C domain.

C, Bar graph showing the intracellular G6P level in indicated RCC4 cells expressing KDM5C mutants.

D, The cell viability in indicated cells treated with 200 μM TBHP. Each three repeats. *P* values were calculated by paired t-test. Data represent means ± SD.

Table S1: The primer sequences of indicated genes using in RT-PCR

Gene	Primer Sequences (5’-3’)
h-GAPDH-RT	F: ACATCAAGAAGGTGGTGAAG
	R: CTGTTGCTGTAGCCAAATTC
h-β-actin-RT	F: CATGTACGTTGCTATCCAGGC
	R: CTCCTTAATGTCACGCACGAT
h-GYS1-RT	F: CTGCTTCCTTGTCCACGTTGA
	R: GGTTGGCAGGCGTTGGACT
h-HK2-RT	F: GGTGGACAGGATACGAGAAAAC
	R: ACATCACATTTCGGAGCCAG
h-PGM1-RT	F: AGCATTCCGTATTTCCAGCAG
	R: GCCAGTTGGGGTCTCATACAAA
h-PPP1R3C-RT	F: ATCCAGGTTTTAGATCCACGTCC
	R: TGTCGTCGTTGAAATTCATCGT
h-G6PD-RT	F: AACATCGCCTGCGTTATCCTC
	R: ACGTCCCGGATGATCCCAA

Table S2: The primer sequences of indicated genes’ promoter using in CHIP-PCR

Gene	Primer sequences (5’-3’)
CHIP-PGM1	F: CGCTCCTCTGCTATTCTCGG
	R: AACCAAGGCAAACTAGGGGG
CHIP-G6PD	F: AGAACCGAGCAGAATCGAGAG
	R: TGACCTGGAGCATGGGAGAT
CHIP-GYS1	F: AATTCGACCTGGAGAACGCA
	R: GTAGCCCCGTCCTCCTACAA
CHIP-HK2	F: GCTCAACCATGACCAAGTGC
	R: CGGCTGCCCAAACTTTTTCC
